# Palliative Care among Heart Failure Patients in Primary Care: A Comparison to Cancer Patients Using English Family Practice Data

**DOI:** 10.1371/journal.pone.0113188

**Published:** 2014-11-25

**Authors:** Amy Gadoud, Eleanor Kane, Una Macleod, Pat Ansell, Steven Oliver, Miriam Johnson

**Affiliations:** 1 Palliative Medicine, Hull York Medical School, University of Hull, Hull, East Yorkshire, United Kingdom; 2 Department of Health Sciences, University of York, York, North Yorkshire, United Kingdom; 3 Primary Care Medicine, Hull York Medical School, University of Hull, Hull, East Yorkshire, United Kingdom; 4 Population Health, Department of Health Sciences and Hull York Medical School, University of York, York, North Yorkshire, United Kingdom; Fondazione G. Monasterio, Italy

## Abstract

**Introduction:**

Patients with heart failure have a significant symptom burden and other palliative care needs often over a longer period than patients with cancer. It is acknowledged that this need may be unmet but by how much has not been quantified in primary care data at the population level.

**Methods:**

This was the first use of Clinical Practice Research Datalink, the world's largest primary care database to explore recognition of the need for palliative care. Heart failure and cancer patients who had died in 2009 aged 18 or over and had at least one year of primary care records were identified. A palliative approach to care among patients with heart failure was compared to that among patients with cancer using entry onto a palliative care register as a marker for a palliative approach to care.

**Results:**

Among patients with heart failure, 7% (234/3 122) were entered on the palliative care register compared to 48% (3 669/7 608) of cancer patients. Of heart failure patients on the palliative care register, 29% (69/234) were entered onto the register within a week of their death.

**Conclusions:**

This confirms that the stark inequity in recognition of palliative care needs for people with heart failure in a large primary care dataset. We recommend a move away from prognosis based criteria for palliative care towards a patient centred approach, with assessment of and attention to palliative needs including advance care planning throughout the disease trajectory.

## Introduction

Early palliative care in cancer consistently leads to better patient and caregiver outcomes [1]. These include improvement in symptoms, quality of life, patient satisfaction, reduced caregiver burden and healthcare costs [2]. Patients with advanced chronic heart failure (CHF) have a significant symptom burden and a range of palliative care needs [3] often over a longer period than patients with cancer [4]. Qualitative studies [3], audits [5], [6] and analysis of data from specialist palliative care services [7], however, suggest unmet palliative care need. Prognosis is variable, both within and between populations and may be difficult to predict in an individual patient [8]–[10].

Position statements from Europe, the United States and Canada agree on the importance of providing a palliative care approach and access to specialist palliative care services for people with heart failure [11]–[13]. Policy initiatives focus on prognosis based criteria such as recognition that the patient is likely to be in the last year of life as the first step in a co-ordinated transition to a palliative approach to care. This approach aims to maximise quality of life by early assessment and addressing of palliative care needs, identifying and supporting patient preferences for care and proactive care planning [14], [15]. The Quality and Outcomes Framework (QOF) is a voluntary incentive scheme for family physicians in the UK [16]. Since 2006 QOF has encouraged primary care to form a practice-based register of all patients, regardless of underlying diagnosis, requiring a palliative approach and to hold multidisciplinary meetings to co-ordinate their care and is used by over 99% of primary care practices in the UK [17]. Separate QOF registers are also held for cancer and heart failure [17]. Family physicians play an important role in palliative care, in most countries, providing care themselves and referring to specialist services and are therefore pivotal to early recognition of palliative care need [18]. In England, palliative care is provided by family physicians and their teams, who can also refer to specialist palliative care services. Specialist palliative care services are available for all life limiting conditions, not just cancer [15].

To the authors' knowledge, this is the first time a national primary care database has been used to investigate if there is any inequity and if present quantify the degree of inequity country-wide with regard to recognition of the need for a palliative approach and to explore the timing of such recognition in relation to the patient' death.

The aim of this study was to explore whether people with CHF are identified as needing a palliative care approach, as marked by entry on the QOF palliative care register and when, in relation to the time of death, this occurred.

## Methods

### Data source

Clinical Practice Research Datalink (CPRD), (previously the General Practice Research Database (GPRD)) is a well-validated database of anonymised electronic contemporaneous medical records from primary care in the UK [19]. It is the world's largest source of anonymous longitudinal data from primary care [20].

Data from five million persons are included in CPRD and demographically it is representative of the UK population as a whole [21]. In the UK the estimated mid 2009 UK population was 62 million [22] and so CPRD represents about 8% of the UK population. Clinical information is recorded in Read codes (coded thesaurus of clinical terms widely used in the NHS especially in primary care).

### Population

We defined the study population as patients that: were registered with a primary care practice that contributes to CPRD database in England; who had died during 2009; were over 18 at the time of their death and had a minimum of one year follow-up records in the database prior to their death that met CPRD acceptability criteria for the quality of data entered. The patient groups (diagnosis of cancer or heart failure) and patients registered as needing a palliative approach to care are QOF indicators and they therefore have defined criteria and Read codes (**see** **table 1** **and Table S1 and S2 in File S1**). Co-morbidities: diabetes mellitus, stroke, chronic kidney disease and chronic obstructive pulmonary disease were also QOF indicators (**see Table S3 to S5 in File S1**). Diseases included on QOF registers are selected on the basis that they are able to be clearly defined and diagnosed [23]–[25]. The data from QOF is being used to calculate prevalence data for chronic disease burden in the population and so are robust methods of identifying a disease [26].

**Table 1:** The QOF criteria and Read codes for: patients identified as needing a palliative care approach, heart failure and cancer.

Disease or health need	Criteria for QOF	Read codes	Medcodes
Identified as needing a palliative approach to care	Clinician predicted survival of less than a year. A positive response to the question–‘would I be surprised if this patient were still alive in 12 months?’ Patients with advanced or irreversible disease and clinical indicators of progressive deterioration such as the Gold Standard Framework Prognostic Indicators Guidance.	1Z01.; 8BA2.; 8BAP.; 8BAS.; 8BAT.; 8BAe.; 8BJ1.; 8CM1.; 8CM4.; 8H6A.; 8H7L.; 8H7g.; 8HH7.; 9EB5.; ZV57C;	7060; 6664; 6924;12739; 18551; 10019; 9755; 74909; 26353; 22288; 26354; 49651; 100607; 100525;
Heart failure	Heart failure is a complex clinical syndrome of symptoms and signs that suggest impairment of the heart as a pump supporting physiological circulation. It is caused by structural or functional abnormalities of the heart. The demonstration of objective evidence of these cardiac abnormalities is necessary for the diagnosis of heart failure to be made.$ All patients with suspected heart failure should be investigated and this is expected to involve, specialist investigation (such as echocardiography and/or natiuretic peptide assay) and often specialist opinion. All heart failure patients, including those with heart failure with preserved ejection fraction should be included.*	G581.%; 585f.; 585g.; G5yy9; G5yyA	398; 884; 2062; 2906; 4024; 1223; 5942; 13189; 18853; 19066; 5255; 32671; 10079; 9524; 17278; 10154; 23707; 27964; 23481; 27884; 51214; 11424; 22262; 43618; 12590; 94870; 101138; 101137
Cancer	All patients with a diagnosis of cancer excluding non-melanoma skin cancers.	B0… - B32z.; B34‥ -B6z0.; Byu‥ - Byu41; Byu5. - ByuE0	#

$ National Clinical Guideline Centre for Acute and Chronic Conditions. NICE Clinical Guideline No 108: CHRONIC HEART FAILURE: National clinical guideline for diagnosis and management in primary and secondary care. London: National Clinical Guideline Centre; 2010.

*As the therapeutic and prognostic benefits of therapy are only proven in those with reduced ejection fraction heart failure, patients with heart failure with preserved ejection fraction may be under represented in heart failure QOF registers.

# Available as a Table S1 in File S1.

Registration on the palliative care register is used as a proxy measure that a palliative approach to care has been recognised.

### Data analysis

The Read codes were translated into medcodes, the coding system used in CPRD and patients divided into: “heart failure only”; “cancer only”; “heart failure and cancer” based on if they were coded as being diagnosed with heart failure or cancer or not at any time prior to death. We extracted the following data: number of patients in each group; number on the palliative care register; time from registration to death; sex. As month of birth is not provided in CPRD, age at death was calculated as from 2009 (year of death) minus the year of birth. Loop diuretic prescriptions were identified using the productcode variable which links to British National Formulary (BNF) chapter headings and so all loop diuretic prescriptions, including combination preparations were identified.

Sensitivity analyses were carried out to determine the effect of only including more recently recorded (within one year and five years of death) heart failure or cancer codes in the analysis as conditions diagnosed in the past may not have contributed to the patient's death. This was performed for all cases and cases where the patient was on the palliative care register. Data is summarised using descriptive statistics, reported as absolute numbers and proportions. The time in days from first recording of clinical code to date of death is presented as median and interquartile ranges and age as mean and standard deviation. Differences in the distributions by age, sex and timing of condition among those on the palliative care register and the total number of patients were tested using Pearson's chi squared. Analyses were conducted using Stata version 12.1.

### Ethical approval

The CPRD Group has obtained ethical approval from a National Research Ethics Service Committee (NRES) for all purely observational research using anonymised CPRD data. This study was approved by the Independent Scientific Advisory Committee (ISAC) for Medicines and Healthcare products Regulatory Agency (MHRA) database research permission (Protocol number: 10_168R). No further ethics approval was required for the analysis of the data as it was purely observational research using anonymised CPRD data.

## Results

A total of 31 667 adult patients in the CPRD database were recorded as having died in 2009 and of these patients 27 689 (87%) had sufficiently complete records to meet the eligibility criteria of at least one year of up to standard records. Of these, 48% were men and the group's mean age was 78 years (standard deviation 14 years). The median year of recording of first clinical information in a record was 1972 (interquartile range 1957 to 1988). 5 311/27 689 (19%) of patients were entered onto the palliative care register prior to their death.

2 811 (90%) of the 3 122 patients with heart failure were recorded as being prescribed loop diuretic therapy. Some loop diuretic prescribing such as those from hospital clinics is not recorded on CPRD. These are unlikely to be many, and would relate to single short periods of prescription as it is unusual for secondary care to take responsibility for on-going diuretic prescription.

Looking at comorbidities of the 27 689 patients 4 740 (17%) had diabetes mellitus, 5050 (18%) had a stroke, 7 537 (27%) had chronic kidney disease and 3 678 (13%) had chronic obstructive pulmonary disease. The heart failure group of 3 122 patients 812 (26%) had diabetes mellitus, 741 (24%) had a stroke, 15 58 (50%) had chronic kidney disease and 603 (19%) had chronic obstructive pulmonary disease. Chronic kidney disease is defined as stage three to five chronic kidney disease, estimated glomerular filtration rate of less than 60 mL/min.

The number of patients by disease register, and the numbers of each also on the palliative care register can be seen in (**table 2**). Comparison of the total population with those on the palliative care register showed cancer patients on the palliative care register tended to be younger than the total but there is no suggestion of age difference among the heart failure patients. There was a marked difference in the percentage of patients on the heart failure or cancer registers who were also on the palliative care register 7% and 48% respectively.

**Table 2:** Proportion of patients, with each diagnosis, identified as being on the palliative care register; demographic data (age, sex) and time from diagnosis recorded until death. Percentages may not total 100 due to rounding.

	Heart failure only		Cancer only		Heart failure and cancer	
	Total; N (column %)	Palliative care register; N (% of Total)	Total; N (column %)	Palliative care register; N (% of Total)	Total; N (column %)	Palliative care register; N (% of Total)
**Total**	3 122 (100)	234 (7)	7 608 (100)	3 669 (48)	803 (100)	257 (32)
**Sex**						
Male	1 462 (47)	110 (47)	3 922 (52)	1 901 (52)	448 (56)	158 (61)
Female	1 660 (53)	124 (53)	3 686 (48)	1 768 (48)	355 (44)	99 (39)
Pearson's χ^2^; p-value	?^2^ = 0.003	p = 0.96	?^2^ = 0.07	p = 0.80	?^2^ = 2.57	p = 0.11
**Age in years at time of death**						
<60	87 (3)	7 (3)	854 (11)	550 (15)	11 (1)	6 (3)
60 to 69	202 (6)	14 (6)	1 492 (20)	872 (24)	57 (7)	24 (9)
70 to 79	598 (19)	44 (19)	2 173 (29)	1 114 (30)	190 (24)	74 (29)
80 to 89	1 382 (44)	111 (47)	2 389 (31)	957 (26)	396 (49)	120 (47)
≥90	853 (27)	58 (25)	700 (9)	176 (5)	149 (19)	33 (13)
Pearson's χ^2^; p-value	?^2^ = 1.13	p = 0.89	?^2^ = 136.7	p<0.01	?^2^ = 8.45	p = 0.08
**Time between first primary care record of condition to death**						
≤1 week	231 (7)	7 (3)	260 (3)	69 (2)	19 (2)	8 (3)
>1 week to 6 weeks	94 (3)	9 (4)	541 (7)	224 (6)	57 (7)	22 (9)
>6 weeks to 6 months	206 (7)	13 (6)	1 282 (17)	705 (19)	128 (16)	53 (21)
>6 months to 1 year	187 (6)	17 (7)	849 (11)	537 (15)	77 (10)	30 (12)
>1 year to 2 years	235 (8)	19 (8)	1 093 (14)	686 (19)	101 (13)	37 (14)
>2 years to 5 years	592 (19)	48 (21)	1 357 (18)	710 (19)	198 (25)	66(26)
>5 years	1 455 (47)	117 (50)	2 109 (28)	706 (19)	219(27)	41(16)
Missing	122 (4)	4 (2)	117 (2)	32 (1)	4 (0.5)	0
Pearson's χ^2^; p-value	?^2^ = 11.1	p = 0.13	?^2^ = 168.0	p<0.01	?^2^ = 16.3	p = 0.02

### Sensitivity analyses

Sensitivity analyses were carried out for the heart failure only and the cancer only groups. The proportions of heart failure patients on the palliative care register with the one year and five year sensitivity analysis were 46/718 (6%) and 113/1 545 (7%) respectively and very similar to the proportion without the sensitivity analysis at 234/3 122 (7%). In the cancer only group the proportion of patients on the palliative care register with the one year and five year sensitivity analysis were similar at 1 535/2 932 (52%) and 2 931/5 382 (54%) but higher than the proportion without sensitivity analysis which was 3 669/7 608 (48%).

### Timing of entry onto palliative care register prior to death

The time from entry onto the palliative care register to the time of death by each diagnosis can be seen in table 3. Of those entered onto the palliative care register, the vast majority of both cancer register and heart failure register groups (79% and 80%) had been entered during the last year of life. Again, in both groups a similar proportion (11% and 8% respectively) had been on the palliative care register for over two years. However, in the heart failure group, a third of patients were not entered onto the palliative care register until the week prior to their death and nearly a half only in the six weeks prior to their death. This contrasts with the cancer patients where only 8% were registered in the week prior to their death, and 29% in the 6 weeks prior to death. The late registration of heart failure patients onto the palliative care register is not explained by late diagnosis of heart failure compared to cancer (table 2). Regardless of when the heart failure diagnosis was recorded the proportion on the palliative care register was the same.

**Table 3:** Time and summary statistics in days (median and interquartile 25 to 75% range) from first time coded as on a palliative care register to date of death for each disease group.

Time between palliative care register and death	Total (%)	Heart failure only N (%)	Cancer only; N (%)	Heart failure and cancer; N (%)
Total	5 311 (100)	234 (100)	3 669(100)	257 (100)
Median (interquartile range)	95 (24–289)	63 (5–220)	115.5 (36–311)	99.5 (28.5–382)
≤1 week	692 (13)	69 (29)	294 (8)	30 (12)
>1 week to 6 weeks	1 113 (21)	40 (17)	755 (21)	61 (24)
>6 weeks to 6 months	1 594 (30)	57 (24)	1 193 (33)	60 (23)
<6 months to 1 year	838 (16)	24 (10)	640 (17)	37 (14)
<1 year to 2 years	664 (12)	17 (7)	504 (14)	32 (12)
>2 years	396 (7)	26 (11)	276 (8)	36 (14)
Missing	14 (0.3)	1 (0.4)	7 (0.2)	1 (0.4)

Percentages may not total 100 due to rounding.

## Discussion

These data show a marked difference between the proportion of heart failure patients (7%) on the palliative care register and that of cancer patients (48%) in a sample representative of national data. Furthermore, patients with heart failure that are entered on the palliative care register are entered much later than those with cancer. Although we present a proxy marker for recognition of the need for a palliative care approach which is specific to the UK health care system, the problem illustrated is likely to one that is familiar in different settings. Barriers to the provision of palliative care for people living with heart failure are described well in the literature [3] [12], [27]. Clinicians are often reluctant to discuss poor prognosis of people with heart failure because of its unpredictable course, concerned they may cause premature alarm and destroy hope. Consequently, these conversations rarely take place [3]. This has been termed “prognostic paralysis” and our findings are consistent with this observation [28].

Our data are similar to English primary care audit data [5] and practice based data, albeit from a smaller study [6]. Data from a Danish primary care population is consistent with increased likelihood of recognition of the need for a palliative approach in cancer patients compared to those with a non-cancer condition; only 4% of chronic obstructive pulmonary disease patients received the free medication provided for those with a terminal illness compared with 55% of patients with lung cancer who died [29].

Our study had the strength of a very large population-based sample drawn from primary care throughout England [19]. The system of health care in the UK is such that the 99% of the population are registered with a family physician [26]. The use of routinely collected data has the advantage that it was not collected for the study, so is representative of actual practice and was not biased by knowledge of the study, especially as it was unselected for the factors of interest. The main search terms are well-defined by QOF thus increasing the confidence that the recording is accurate, and likely to be complete or near complete by family physicians. Ninety per cent of the heart failure decedents were prescribed loop diuretics. The QOF also encouraged prescribing of an ACE inhibitor or Angiotensin Receptor Blocker to 40 to 80% of patients on the register, which was achieved by 97.7% of practices in 2009. Additional prescribing of a B-blocker to 40 to 60% of patients on the register was achieved by 96.1% of practices [30].

Heart failure has a variable disease trajectory and sudden death may occur. From the data recorded in CPRD it is not possible to identify which deaths were sudden. It is estimated that each GP caseload in the UK has about 20 deaths (all cause) per year, but only one or two of these deaths are sudden.(10) Another source suggests that about a quarter of deaths are sudden.(3) These sudden deaths are due to all causes, not just heart failure patients. However if we over-estimated the number of sudden deaths as a quarter of heart failure deaths, and exclude from the analysis, this would still mean that only 10% (234/2341.5) of heart failure patients were recognised as needing palliative care prior to their death.

About 20 per cent of patients were on the palliative care register for more than a year. Some practices may use a problem based approach to palliative care and so have patients who are not in the last year of life or alternatively it could be due to difficulty in accurately predicting the last year of life.

### Limitations

Registration on the palliative care QOF is a proxy measure for clinical recognition of the need for a palliative approach to care and it may be less useful in palliative care for people with non-malignant conditions. Despite clear policy guidance, there may be a perception that the palliative care QOF is for cancer patients only and that those with other conditions do not need this approach. However, if so, this in itself is an inequity. Identification is only the first step of a palliative care approach. As we used in-life diagnosis data rather than cause of death data, such as death certification, our denominator may be overestimated. However, death certificates may be inaccurate and may fail to record the contribution of an underlying condition such as cancer or heart failure [31]. Our sensitivity analyses using diagnoses within a year and five years of death did not indicate that this was a major problem for heart failure. For cancer there was a higher percentage in the sensitivity analysis indicating that the proportion of cancer patients identified as needing a palliative care approach may be higher than reported and the inequity between the two groups, may be wider. In addition, central death certificate data from all deaths registered in England during 2009, the same year as this study, showed the proportions dying from chronic coronary heart disease (14% deaths) and cancer (29% deaths) are similar to this CPRD study.

### Implications for practice, policy or future research

We report the first use, to our knowledge, of the world's largest primary care database using nationally representative data to quantify the recognition of the need for a palliative approach to care and its timing in relation to death. This method could be used to explore other progressive life limiting illnesses such as chronic obstructive airway disease and dementia.

Recognition of the need for a palliative approach to care tends to be done much later in the disease trajectory in those with heart failure. Nearly one in three heart failure patients in our dataset were first registered within a week of death. This is a time when inevitable and irreversible deterioration is more apparent, but may be more difficult to ascertain a patient's wishes during such rapid deterioration.

A palliative care approach can and should run in parallel with optimal heart failure management [11], [12]. Awareness for clinicians and patients of an approach which can incorporate palliative care as needed from diagnosis and throughout the disease trajectory will prevent the difficulties of an all or none transition point or the prognostic paralysis that occurs when “the right time” becomes the primary focus [12], [27], [32]. As with cancer, many people with heart failure do wish to discuss end-of-life issues [3] and guidance and training have been developed that allow for discussions about poor prognosis and decisions about future care despite uncertain disease trajectories [27].

Prognostication regarding the last year of life is heart failure is very difficult. The Gold Standards Framework-Prognostic Indicator Guide [33], recommended as a tool to predict which patients should be on the QOF palliative care register, but has no published data to support its accuracy [34]. A recent study has shown that neither the Gold Standards Framework-Prognostic Indicator Guide or the Seattle Heart Failure Model (an extensively validated tool, considered ‘gold standard’ for routine prognostication in ambulant heart failure populations [34]) were useful in accurately predicting the last year of life in a group of community heart failure patients in the UK [33]. We suggest that future research regarding identification of patients for a palliative care approach for patients with heart failure should focus on patients' problems rather than prognosis. This will allow a more patient centred approach that can facilitate conversations about future even if that future is not yet certain.

After more than a decade of national policy recommending a palliative care for patients with heart failure, the stark finding of this large UK primary care database study was that people with heart failure are relatively absent from the recommended primary care system for co-ordinating palliative care. This has implications for individuals and their families. It also has the globally applicable lesson to move away from a prognosis based criteria for palliative care which discriminate against progressive, life limiting illnesses with an uncertain prognosis. We recommend patient centred approaches that include an integrated approach to palliative care provision in heart failure, with assessment of and attention to palliative needs including advance care planning throughout the disease trajectory.

## Supporting Information

File S1 Contains the following files: **Table S1.** Medcodes for being on the cancer register. **Table S2.** Medcodes for being on the heart failure register. **Table S3.** Medcodes for being on the diabetes register. **Table S4.** Medcodes for being on the stroke register. **Table S5.** Medcodes for being on the COPD register.
(DOCX)
