# Supplementary material for: Palliative Care among Heart Failure Patients in Primary Care: A Comparison to Cancer Patients Using English Family Practice Data
Source: PLoS One. 2014 Nov 25;9(11):e113188. doi: 10.1371/journal.pone.0113188 (PMC4244094; doi:10.1371/journal.pone.0113188)
Supplement: File S1 — Contains the following files: Table S1. Medcodes for being on the cancer register. Table S2. Medcodes for being on the heart failure register. Table S3. Medcodes for being on the diabetes register. Table S4. Medcodes for being on the stroke register. Table S5. Medcodes for being on the COPD register. (DOCX) [file pone.0113188.s001.docx]

Table of medcodes

| **medcode** | **readcode** | **readterm** |
| --- | --- | --- |
| 318 | B210.00 | Malignant neoplasm of glottis |
| 319 | B21..00 | Malignant neoplasm of larynx |
| 348 | B34..11 | Ca female breast |
| 779 | B49..00 | Malignant neoplasm of urinary bladder |
| 780 | B46..00 | Malignant neoplasm of prostate |
| 782 | B....00 | Neoplasms |
| 865 | B32..00 | Malignant melanoma of skin |
| 1056 | B5z..00 | Malignant neoplasm of other and unspecified site NOS |
| 1062 | B10..00 | Malignant neoplasm of oesophagus |
| 1220 | B13..00 | Malignant neoplasm of colon |
| 1481 | B600.00 | Reticulosarcoma |
| 1599 | B4A0.00 | Malignant neoplasm of kidney parenchyma |
| 1800 | B141.00 | Malignant neoplasm of rectum |
| 1952 | B580.00 | Secondary malignant neoplasm of kidney |
| 1986 | B440.11 | Cancer of ovary |
| 2462 | B61..00 | Hodgkin's disease |
| 2587 | B22z.11 | Lung cancer |
| 2744 | B40..00 | Malignant neoplasm of uterus, part unspecified |
| 2747 | B41..00 | Malignant neoplasm of cervix uteri |
| 2755 | B....11 | Cancers |
| 2815 | B133.00 | Malignant neoplasm of sigmoid colon |
| 2890 | B430200 | Malignant neoplasm of endometrium of corpus uteri |
| 2961 | B47z.11 | Seminoma of testis |
| 3213 | B430.00 | Malignant neoplasm of corpus uteri, excluding isthmus |
| 3230 | B41..11 | Cervical carcinoma (uterus) |
| 3357 | B1...11 | Carcinoma of digestive organs and peritoneum |
| 3541 | B48..00 | Malignant neoplasm of penis and other male genital organs |
| 3604 | B627.00 | Non - Hodgkin's lymphoma |
| 3811 | B134.00 | Malignant neoplasm of caecum |
| 3903 | B22z.00 | Malignant neoplasm of bronchus or lung NOS |
| 3968 | B34..00 | Malignant neoplasm of female breast |
| 4072 | B680.00 | Acute leukaemia NOS |
| 4137 | B570.00 | Secondary malignant neoplasm of lung |
| 4218 | B541.00 | Malignant neoplasm of parathyroid gland |
| 4222 | B64..11 | Lymphatic leukaemia |
| 4250 | B68z.00 | Leukaemia NOS |
| 4251 | B640.00 | Acute lymphoid leukaemia |
| 4388 | B020.00 | Malignant neoplasm of parotid gland |
| 4403 | B577.11 | Liver metastases |
| 4413 | B650.00 | Acute myeloid leukaemia |
| 4554 | B454.00 | Malignant neoplasm of vulva unspecified |
| 4555 | B45..00 | Malig neop of other and unspecified female genital organs |
| 4865 | B10z.11 | Oesophageal cancer |
| 4870 | B625.11 | Histiocytosis X (acute, progressive) |
| 4944 | B630.00 | Multiple myeloma |
| 5062 | B30..11 | Chondroma |
| 5137 | B624.11 | Leukaemic reticuloendotheliosis |
| 5179 | B620.00 | Nodular lymphoma (Brill - Symmers disease) |
| 5198 | B583000 | Secondary malignant neoplasm of brain |
| 5199 | B583200 | Cerebral metastasis |
| 5637 | B53..00 | Malignant neoplasm of thyroid gland |
| 5842 | B58..00 | Secondary malignant neoplasm of other specified sites |
| 5901 | B141.12 | Rectal carcinoma |
| 6115 | B6y0.00 | Myeloproliferative disorder |
| 6170 | B590.11 | Carcinomatosis |
| 6471 | B57..11 | Metastases of respiratory and/or digestive systems |
| 6701 | B565.00 | Secondary and unspec malig neop intrapelvic lymph nodes |
| 6806 | B12..00 | Malignant neoplasm of small intestine and duodenum |
| 6935 | B131.00 | Malignant neoplasm of transverse colon |
| 7046 | B43..00 | Malignant neoplasm of body of uterus |
| 7176 | B65..00 | Myeloid leukaemia |
| 7219 | B141.11 | Carcinoma of rectum |
| 7484 | B226.00 | Mesothelioma |
| 7654 | B585.00 | Secondary malignant neoplasm of bone and bone marrow |
| 7740 | B470200 | Seminoma of undescended testis |
| 7805 | B440.00 | Malignant neoplasm of ovary |
| 7830 | B56..11 | Lymph node metastases |
| 7940 | ByuDF11 | [X]Non-Hodgkin's lymphoma NOS |
| 7978 | B4A0000 | Hypernephroma |
| 7982 | B161200 | Malignant neoplasm of common bile duct |
| 8154 | B576200 | Malignant ascites |
| 8166 | B17..00 | Malignant neoplasm of pancreas |
| 8386 | B11..00 | Malignant neoplasm of stomach |
| 8550 | B542000 | Malignant neoplasm of pituitary gland |
| 8625 | B641.00 | Chronic lymphoid leukaemia |
| 8649 | ByuDF00 | [X]Non-Hodgkin's lymphoma, unspecified type |
| 8693 | B5...11 | Carcinoma of other and unspecified sites |
| 8771 | B170.00 | Malignant neoplasm of head of pancreas |
| 8918 | B15..00 | Malignant neoplasm of liver and intrahepatic bile ducts |
| 9030 | B55..00 | Malignant neoplasm of other and ill-defined sites |
| 9088 | B130.00 | Malignant neoplasm of hepatic flexure of colon |
| 9118 | B13z.11 | Colonic cancer |
| 9237 | B21z.00 | Malignant neoplasm of larynx NOS |
| 9470 | B34z.00 | Malignant neoplasm of female breast NOS |
| 9476 | B471100 | Teratoma of descended testis |
| 9491 | B142.11 | Anal carcinoma |
| 9505 | B582600 | Secondary malignant neoplasm of skin of breast |
| 9600 | B232.00 | Mesothelioma of pleura |
| 9618 | B56..00 | Secondary and unspecified malignant neoplasm of lymph nodes |
| 9622 | B525.00 | Malignant neoplasm of cauda equina |
| 9902 | B3...11 | Carcinoma of bone, connective tissue, skin and breast |
| 9984 | B00..11 | Carcinoma of lip |
| 10283 | B01..00 | Malignant neoplasm of tongue |
| 10314 | B057.00 | Overlapping lesion of other and unspecified parts of mouth |
| 10358 | B222.00 | Malignant neoplasm of upper lobe, bronchus or lung |
| 10368 | B11..11 | Gastric neoplasm |
| 10698 | B450100 | Malignant neoplasm of vaginal vault |
| 10726 | B651.00 | Chronic myeloid leukaemia |
| 10851 | B51..11 | Cerebral tumour - malignant |
| 10864 | B132.00 | Malignant neoplasm of descending colon |
| 10946 | B136.00 | Malignant neoplasm of ascending colon |
| 10949 | B162.00 | Malignant neoplasm of ampulla of Vater |
| 10995 | B5...00 | Malignant neoplasm of other and unspecified sites |
| 11009 | B1z..00 | Malig neop oth/ill-defined sites digestive tract/peritoneum |
| 11035 | B593.00 | Primary malignant neoplasm of unknown site |
| 11628 | B1z0.11 | Cancer of bowel |
| 11991 | B454.11 | Primary vulval cancer |
| 12006 | B621.00 | Mycosis fungoides |
| 12323 | B6...00 | Malignant neoplasm of lymphatic and haemopoietic tissue |
| 12335 | B62y.00 | Malignant lymphoma NOS |
| 12389 | B4A1.00 | Malignant neoplasm of renal pelvis |
| 12464 | B62x200 | Peripheral T-cell lymphoma |
| 12490 | B550200 | Malignant neoplasm of nose NOS |
| 12499 | Byu6.00 | [X]Malignant neoplasm of breast |
| 12539 | B3...12 | Sarcoma of bone and connective tissue |
| 12582 | B224100 | Malignant neoplasm of lower lobe of lung |
| 12870 | B221.00 | Malignant neoplasm of main bronchus |
| 13243 | B22..00 | Malignant neoplasm of trachea, bronchus and lung |
| 13252 | B4...00 | Malignant neoplasm of genitourinary organ |
| 13559 | B4A..00 | Malig neop of kidney and other unspecified urinary organs |
| 13569 | B590.00 | Disseminated malignancy NOS |
| 14712 | B00..00 | Malignant neoplasm of lip |
| 14792 | B05..00 | Malignant neoplasm of other and unspecified parts of mouth |
| 14800 | B11z.00 | Malignant neoplasm of stomach NOS |
| 15027 | B62yz00 | Malignant lymphoma NOS |
| 15036 | B626.00 | Malignant mast cell tumours |
| 15103 | B577.00 | Secondary malignant neoplasm of liver |
| 15148 | B47..00 | Malignant neoplasm of testis |
| 15182 | B31z.00 | Malignant neoplasm of connective and soft tissue, site NOS |
| 15211 | B630.12 | Myelomatosis |
| 15221 | B220.00 | Malignant neoplasm of trachea |
| 15223 | B4A2.00 | Malignant neoplasm of ureter |
| 15504 | B62y800 | Malignant lymphoma NOS of lymph nodes of multiple sites |
| 15507 | B56z.00 | Secondary and unspec malig neop lymph nodes NOS |
| 15644 | B4A3.00 | Malignant neoplasm of urethra |
| 15684 | B204.00 | Malignant neoplasm of frontal sinus |
| 15709 | B1...00 | Malignant neoplasm of digestive organs and peritoneum |
| 15711 | B510.00 | Malignant neoplasm cerebrum (excluding lobes and ventricles) |
| 15907 | B16z.00 | Malignant neoplasm gallbladder/extrahepatic bile ducts NOS |
| 15976 | B552.00 | Malignant neoplasm of abdomen |
| 15989 | B47z.12 | Teratoma of testis |
| 15991 | B506.00 | Malignant neoplasm of choroid |
| 16075 | B30z.00 | Malignant neoplasm of bone and articular cartilage NOS |
| 16105 | B160.00 | Malignant neoplasm of gallbladder |
| 16126 | B150000 | Primary carcinoma of liver |
| 16213 | B572.00 | Secondary malignant neoplasm of pleura |
| 16241 | B060.00 | Malignant neoplasm of tonsil |
| 16280 | B550400 | Malignant neoplasm of neck NOS |
| 16297 | B0z0.00 | Malignant neoplasm of pharynx unspecified |
| 16298 | B18z.00 | Malignant neoplasm of retroperitoneum and peritoneum NOS |
| 16416 | B681.00 | Chronic leukaemia NOS |
| 16500 | B58z.00 | Secondary malignant neoplasm of other specified site NOS |
| 16704 | B302.00 | Malignant neoplasm of vertebral column |
| 16760 | B58y000 | Secondary malignant neoplasm of breast |
| 16874 | B4...11 | Carcinoma of genitourinary organ |
| 16915 | B151.00 | Malignant neoplasm of intrahepatic bile ducts |
| 16967 | B432.00 | Malignant neoplasm of overlapping lesion of corpus uteri |
| 17056 | B6y0.11 | Myeloproliferative disease |
| 17182 | B627C11 | Follicular lymphoma NOS |
| 17391 | B221000 | Malignant neoplasm of carina of bronchus |
| 17460 | B627700 | Diffuse non-Hodgkin's lymphoblastic (diffuse) lymphoma |
| 17475 | B300A00 | Malignant neoplasm of maxilla |
| 17559 | B1z0.00 | Malignant neoplasm of intestinal tract, part unspecified |
| 17841 | B481.00 | Malignant neoplasm of glans penis |
| 17874 | B181.00 | Mesothelioma of peritoneum |
| 17887 | B62x.00 | Malignant lymphoma otherwise specified |
| 17912 | B042.00 | Malignant neoplasm, overlapping lesion of floor of mouth |
| 18231 | B540.11 | Phaeochromocytoma |
| 18314 | B30..00 | Malignant neoplasm of bone and articular cartilage |
| 18608 | B3...00 | Malig neop of bone, connective tissue, skin and breast |
| 18613 | B120.00 | Malignant neoplasm of duodenum |
| 18616 | B58y.00 | Secondary malignant neoplasm of other specified sites |
| 18617 | B51..00 | Malignant neoplasm of brain |
| 18619 | B137.00 | Malignant neoplasm of splenic flexure of colon |
| 18632 | B135.00 | Malignant neoplasm of appendix |
| 18658 | B562300 | Secondary and unspec malig neop common iliac lymph nodes |
| 18676 | B585000 | Pathological fracture due to metastatic bone disease |
| 18678 | B224000 | Malignant neoplasm of lower lobe bronchus |
| 18712 | B4A..11 | Renal malignant neoplasm |
| 18882 | B006.00 | Malignant neoplasm of overlapping lesion of lip |
| 19028 | B630100 | Solitary myeloma |
| 19140 | B614800 | Hodgkin's nodular sclerosis of lymph nodes of multiple sites |
| 19141 | B44..00 | Malignant neoplasm of ovary and other uterine adnexa |
| 19144 | Byu4.00 | [X]Melanoma and other malignant neoplasms of skin |
| 19162 | B493.00 | Malignant neoplasm of anterior wall of urinary bladder |
| 19226 | B513.00 | Malignant neoplasm of parietal lobe |
| 19318 | B112.00 | Malignant neoplasm of pyloric antrum of stomach |
| 19321 | B311300 | Malignant neoplasm of connective and soft tissue of hand |
| 19372 | B64..00 | Lymphoid leukaemia |
| 19389 | B3y..00 | Malig neop of bone, connective tissue, skin and breast OS |
| 19415 | B0...00 | Malignant neoplasm of lip, oral cavity and pharynx |
| 19423 | B35..00 | Malignant neoplasm of male breast |
| 19437 | B30z000 | Osteosarcoma |
| 19444 | Byu4100 | [X]Malignant melanoma of skin, unspecified |
| 19475 | B471.00 | Malignant neoplasm of descended testis |
| 19945 | B582.00 | Secondary malignant neoplasm of skin |
| 19974 | B660.00 | Acute monocytic leukaemia |
| 20092 | B04..00 | Malignant neoplasm of floor of mouth |
| 20159 | B56y.00 | Secondary and unspec malig neop lymph nodes multiple sites |
| 20160 | B50..00 | Malignant neoplasm of eye |
| 20166 | B45z.00 | Malignant neoplasm of female genital organ NOS |
| 20170 | B222.11 | Pancoast's syndrome |
| 20292 | B02..00 | Malignant neoplasm of major salivary glands |
| 20440 | B69..00 | Myelomonocytic leukaemia |
| 20685 | B346.00 | Malignant neoplasm of axillary tail of female breast |
| 21329 | B630200 | Plasmacytoma NOS |
| 21330 | B180.00 | Malignant neoplasm of retroperitoneum |
| 21402 | B602.00 | Burkitt's lymphoma |
| 21549 | B627C00 | Follicular non-Hodgkin's lymphoma |
| 21590 | B58y500 | Secondary malignant neoplasm of prostate |
| 21620 | B111.00 | Malignant neoplasm of pylorus of stomach |
| 21698 | B221z00 | Malignant neoplasm of main bronchus NOS |
| 21715 | Byu5011 | [X]Mesothelioma of lung |
| 21786 | B471000 | Seminoma of descended testis |
| 22050 | B691.00 | Chronic myelomonocytic leukaemia |
| 22146 | B581100 | Secondary malignant neoplasm of bladder |
| 22158 | B630000 | Malignant plasma cell neoplasm, extramedullary plasmacytoma |
| 22163 | B134.11 | Carcinoma of caecum |
| 22187 | B150300 | Hepatocellular carcinoma |
| 22290 | B313.00 | Malignant neoplasm of connective and soft tissue of thorax |
| 22441 | B212.00 | Malignant neoplasm of subglottis |
| 22524 | B58yz00 | Secondary malignant neoplasm of other specified site NOS |
| 22893 | B06..00 | Malignant neoplasm of oropharynx |
| 22894 | B110100 | Malignant neoplasm of cardio-oesophageal junction of stomach |
| 23380 | B340000 | Malignant neoplasm of nipple of female breast |
| 23389 | B200.00 | Malignant neoplasm of nasal cavities |
| 23399 | B344.00 | Malignant neoplasm of upper-outer quadrant of female breast |
| 23433 | B161.00 | Malignant neoplasm of extrahepatic bile ducts |
| 23861 | B551100 | Malignant neoplasm of chest wall NOS |
| 24048 | B180200 | Malignant neoplasm of retrocaecal tissue |
| 24235 | B524.00 | Malig neopl peripheral nerves and autonomic nervous system |
| 24301 | B57..12 | Secondary carcinoma of respiratory and/or digestive systems |
| 24370 | B142.00 | Malignant neoplasm of anal canal |
| 24374 | B0...11 | Carcinoma of lip, oral cavity and pharynx |
| 24397 | B061.00 | Malignant neoplasm of tonsillar fossa |
| 24456 | B201.00 | Malig neop auditory tube, middle ear and mastoid air cells |
| 24675 | B07..00 | Malignant neoplasm of nasopharynx |
| 24852 | B016.00 | Malignant neoplasm of lingual tonsil |
| 25191 | B68..00 | Leukaemia of unspecified cell type |
| 25366 | B561300 | Secondary and unspec malig neop ant mediastinal lymph nodes |
| 25535 | B150.00 | Primary malignant neoplasm of liver |
| 25602 | B326400 | Malignant melanoma of finger |
| 25886 | B222100 | Malignant neoplasm of upper lobe of lung |
| 26034 | B591.00 | Other malignant neoplasm NOS |
| 26134 | B064000 | Malignant neoplasm of epiglottis, free border |
| 26165 | B211.00 | Malignant neoplasm of supraglottis |
| 26393 | B152.00 | Malignant neoplasm of liver unspecified |
| 26448 | B060000 | Malignant neoplasm of faucial tonsil |
| 26454 | B45X.00 | Malignant neoplasm/overlapping lesion/feml genital organs |
| 26652 | B20..00 | Malig neop nasal cavities, middle ear and accessory sinuses |
| 26813 | B21y.00 | Malignant neoplasm of larynx, other specified site |
| 26853 | B340.00 | Malignant neoplasm of nipple and areola of female breast |
| 27330 | B624.00 | Leukaemic reticuloendotheliosis |
| 27340 | B670.11 | Di Guglielmo's disease |
| 27391 | B576100 | Secondary malignant neoplasm of peritoneum |
| 27416 | B601.00 | Lymphosarcoma |
| 27449 | B554.00 | Malignant neoplasm of upper limb NOS |
| 27458 | B661.00 | Chronic monocytic leukaemia |
| 27483 | B240.00 | Malignant neoplasm of thymus |
| 27520 | B651z00 | Chronic myeloid leukaemia NOS |
| 27528 | B303.00 | Malignant neoplasm of ribs, sternum and clavicle |
| 27540 | B4A1000 | Malignant neoplasm of renal calyces |
| 27617 | B45y000 | Malignant neoplasm of overlapping lesion of vulva |
| 27651 | B58..11 | Secondary carcinoma of other specified sites |
| 27664 | B65y100 | Acute promyelocytic leukaemia |
| 27715 | B242.00 | Malignant neoplasm of anterior mediastinum |
| 27790 | B641.11 | Chronic lymphatic leukaemia |
| 27855 | B140.00 | Malignant neoplasm of rectosigmoid junction |
| 27897 | B143.00 | Malignant neoplasm of anus unspecified |
| 28003 | B420.00 | Choriocarcinoma |
| 28059 | B560600 | Secondary and unspec malig neop of facial lymph nodes |
| 28069 | B505.00 | Malignant neoplasm of retina |
| 28148 | B540.00 | Malignant neoplasm of adrenal gland |
| 28163 | B13z.00 | Malignant neoplasm of colon NOS |
| 28241 | B496.00 | Malignant neoplasm of ureteric orifice |
| 28276 | B675.00 | Acute myelofibrosis |
| 28311 | B41z.00 | Malignant neoplasm of cervix uteri NOS |
| 28451 | B08z.00 | Malignant neoplasm of hypopharynx NOS |
| 28556 | B32z.00 | Malignant melanoma of skin NOS |
| 28559 | B055z00 | Malignant neoplasm of palate NOS |
| 28639 | B627000 | Follicular non-Hodgkin's small cleaved cell lymphoma |
| 28665 | B07z.00 | Malignant neoplasm of nasopharynx NOS |
| 28727 | B575000 | Secondary malignant neoplasm of colon |
| 28919 | B521.00 | Malignant neoplasm of cerebral meninges |
| 29160 | B313000 | Malignant neoplasm of connective and soft tissue of axilla |
| 29178 | B614.00 | Hodgkin's disease, nodular sclerosis |
| 29283 | B2zy.00 | Malignant neoplasm of other site of respiratory tract |
| 29462 | B4Az.00 | Malignant neoplasm of kidney or urinary organs NOS |
| 29735 | B30..12 | Osteoma |
| 29826 | B342.00 | Malignant neoplasm of upper-inner quadrant of female breast |
| 29876 | B613z00 | Hodgkin's, lymphocytic-histiocytic predominance NOS |
| 30165 | B18y200 | Malignant neoplasm of mesorectum |
| 30402 | B050.11 | Malignant neoplasm of buccal mucosa |
| 30511 | B54..00 | Malig neop of other endocrine glands and related structures |
| 30526 | Byu5100 | [X]Mesothelioma, unspecified |
| 30542 | B312300 | Malig neop of connective and soft tissue of lower leg |
| 30632 | B67z.00 | Other specified leukaemia NOS |
| 30646 | B6y..00 | Malignant neoplasm lymphatic or haematopoietic tissue OS |
| 30700 | B10z.00 | Malignant neoplasm of oesophagus NOS |
| 31102 | B49z.00 | Malignant neoplasm of urinary bladder NOS |
| 31188 | B224.00 | Malignant neoplasm of lower lobe, bronchus or lung |
| 31210 | B150100 | Hepatoblastoma of liver |
| 31268 | B223.00 | Malignant neoplasm of middle lobe, bronchus or lung |
| 31324 | B626800 | Mast cell malignancy of lymph nodes of multiple sites |
| 31364 | B050.00 | Malignant neoplasm of cheek mucosa |
| 31393 | B160.11 | Carcinoma gallbladder |
| 31399 | B555.00 | Malignant neoplasm of lower limb NOS |
| 31546 | B341.00 | Malignant neoplasm of central part of female breast |
| 31573 | B23..00 | Malignant neoplasm of pleura |
| 31576 | B627B00 | Other types of follicular non-Hodgkin's lymphoma |
| 31586 | B64y100 | Prolymphocytic leukaemia |
| 31608 | B43y.00 | Malignant neoplasm of other site of uterine body |
| 31700 | B222000 | Malignant neoplasm of upper lobe bronchus |
| 31701 | B651.11 | Chronic granulocytic leukaemia |
| 31794 | B627W00 | Unspecified B-cell non-Hodgkin's lymphoma |
| 32022 | B110.00 | Malignant neoplasm of cardia of stomach |
| 32024 | B030.00 | Malignant neoplasm of upper gum |
| 32174 | B202.00 | Malignant neoplasm of maxillary sinus |
| 32362 | B113.00 | Malignant neoplasm of fundus of stomach |
| 32372 | B302100 | Malignant neoplasm of thoracic vertebra |
| 32768 | B325100 | Malignant melanoma of breast |
| 32955 | B41y.00 | Malignant neoplasm of other site of cervix |
| 33333 | B62..00 | Other malignant neoplasm of lymphoid and histiocytic tissue |
| 33344 | B65z.00 | Myeloid leukaemia NOS |
| 33388 | B071000 | Malignant neoplasm of adenoid |
| 33395 | B560200 | Secondary and unspec malig neop superficial cervical LN |
| 33444 | B221100 | Malignant neoplasm of hilus of lung |
| 33617 | B43z.00 | Malignant neoplasm of body of uterus NOS |
| 33833 | B301.00 | Malignant neoplasm of mandible |
| 33843 | B583.00 | Secondary malignant neoplasm of brain and spinal cord |
| 33871 | B122.00 | Malignant neoplasm of ileum |
| 34012 | B08..00 | Malignant neoplasm of hypopharynx |
| 34075 | B2...00 | Malig neop of respiratory tract and intrathoracic organs |
| 34089 | B62y400 | Malignant lymphoma NOS of lymph nodes of axilla and arm |
| 34145 | B58y600 | Secondary malignant neoplasm of testis |
| 34259 | B325300 | Malignant melanoma of groin |
| 34388 | B17z.00 | Malignant neoplasm of pancreas NOS |
| 34409 | B010000 | Malignant neoplasm of base of tongue dorsal surface |
| 34451 | B31..00 | Malignant neoplasm of connective and other soft tissue |
| 34692 | B68y.00 | Other leukaemia of unspecified cell type |
| 34742 | B23z.00 | Malignant neoplasm of pleura NOS |
| 34878 | B308300 | Malignant neoplasm of medial cuneiform |
| 34926 | B625.00 | Letterer-Siwe disease |
| 35014 | B622.00 | Sezary's disease |
| 35039 | B163.00 | Malignant neoplasm, overlapping lesion of biliary tract |
| 35053 | B57..00 | Secondary malig neop of respiratory and digestive systems |
| 35113 | Byu9.00 | [X]Malignant neoplasm of urinary tract |
| 35180 | Byu1.00 | [X]Malignant neoplasm of digestive organs |
| 35186 | ByuC.00 | [X]Malignant neoplasm of ill-defined, secondary and unspeci |
| 35285 | ByuA.00 | [X]Malignant neoplasm of eye, brain and other parts of cent |
| 35325 | Byu2.00 | [X]Malignant neoplasm of respiratory and intrathoracic orga |
| 35357 | B14..00 | Malignant neoplasm of rectum, rectosigmoid junction and anus |
| 35364 | B576000 | Secondary malignant neoplasm of retroperitoneum |
| 35535 | B173.00 | Malignant neoplasm of pancreatic duct |
| 35795 | B174.00 | Malignant neoplasm of Islets of Langerhans |
| 35875 | B66..00 | Monocytic leukaemia |
| 35963 | B492.00 | Malignant neoplasm of lateral wall of urinary bladder |
| 35999 | B582200 | Secondary malignant neoplasm of skin of neck |
| 36147 | B153.00 | Secondary malignant neoplasm of liver |
| 36161 | B012.00 | Malignant neoplasm of tongue, tip and lateral border |
| 36200 | B575z00 | Secondary malig neop of large intestine or rectum NOS |
| 36325 | B470300 | Teratoma of undescended testis |
| 36371 | B225.00 | Malignant neoplasm of overlapping lesion of bronchus & lung |
| 36401 | B587.00 | Secondary malignant neoplasm of adrenal gland |
| 36495 | B161211 | Carcinoma common bile duct |
| 36716 | B04z.00 | Malignant neoplasm of floor of mouth NOS |
| 36899 | B327800 | Malignant melanoma of toe |
| 36949 | B49y.00 | Malignant neoplasm of other site of urinary bladder |
| 37096 | B015.00 | Malignant neoplasm of tongue, junctional zone |
| 37112 | B6...11 | Malignant neoplasm of histiocytic tissue |
| 37182 | B63..00 | Multiple myeloma and immunoproliferative neoplasms |
| 37272 | B67..00 | Other specified leukaemia |
| 37328 | B450.00 | Malignant neoplasm of vagina |
| 37461 | B64y200 | Adult T-cell leukaemia |
| 37468 | B671.00 | Chronic erythraemia |
| 37516 | B054.00 | Malignant neoplasm of uvula |
| 37540 | B563000 | Secondary and unspec malig neop axillary lymph nodes |
| 37549 | B05z000 | Kaposi's sarcoma of palate |
| 37553 | B007.00 | Malignant neoplasm of lip, unspecified |
| 37590 | B052.00 | Malignant neoplasm of hard palate |
| 37618 | B551000 | Malignant neoplasm of axilla NOS |
| 37724 | B056.00 | Malignant neoplasm of retromolar area |
| 37805 | B213100 | Malignant neoplasm of cricoid cartilage |
| 37810 | B220z00 | Malignant neoplasm of trachea NOS |
| 37842 | B303000 | Malignant neoplasm of rib |
| 37859 | B110z00 | Malignant neoplasm of cardia of stomach NOS |
| 37872 | B327400 | Malignant melanoma of lower leg |
| 37916 | B05y.00 | Malignant neoplasm of other specified mouth parts |
| 37919 | B561000 | Secondary and unspec malig neop internal mammary lymph nodes |
| 37940 | B072000 | Malignant neoplasm of pharyngeal recess |
| 38005 | B621z00 | Mycosis fungoides NOS |
| 38331 | B64yz00 | Other lymphoid leukaemia NOS |
| 38343 | B560700 | Secondary and unspec malig neop submental lymph nodes |
| 38475 | B34yz00 | Malignant neoplasm of other site of female breast NOS |
| 38488 | B013z00 | Malignant neoplasm of ventral tongue surface NOS |
| 38510 | B47z.00 | Malignant neoplasm of testis NOS |
| 38689 | B325.00 | Malignant melanoma of trunk (excluding scrotum) |
| 38736 | B5y..00 | Malignant neoplasm of other and unspecified site OS |
| 38862 | B490.00 | Malignant neoplasm of trigone of urinary bladder |
| 38914 | B64z.00 | Lymphoid leukaemia NOS |
| 38918 | B583100 | Secondary malignant neoplasm of spinal cord |
| 38931 | B4y..00 | Malignant neoplasm of genitourinary organ OS |
| 38938 | B306z00 | Malignant neoplasm of pelvis, sacrum or coccyx NOS |
| 38939 | B613.00 | Hodgkin's disease, lymphocytic-histiocytic predominance |
| 38961 | B22y.00 | Malignant neoplasm of other sites of bronchus or lung |
| 38978 | B15z.00 | Malignant neoplasm of liver and intrahepatic bile ducts NOS |
| 39027 | ByuC000 | [X]Malignant neoplasm of other specified sites |
| 39084 | B0z2.00 | Malignant neoplasm of laryngopharynx |
| 39088 | B514.00 | Malignant neoplasm of occipital lobe |
| 39187 | B631.00 | Plasma cell leukaemia |
| 39336 | B6y1.00 | Myelosclerosis with myeloid metaplasia |
| 39413 | B18y500 | Malignant neoplasm of pelvic peritoneum |
| 39430 | B0zz.00 | Malignant neoplasm of lip, oral cavity and pharynx NOS |
| 39433 | B560500 | Secondary and unspec malig neop submandibular lymph nodes |
| 39531 | B25..00 | Malig neo, overlapping lesion of heart, mediastinum & pleura |
| 39554 | B063.00 | Malignant neoplasm of vallecula |
| 39590 | B206.00 | Malignant neoplasm, overlapping lesion of accessory sinuses |
| 39629 | B653100 | Granulocytic sarcoma |
| 39798 | B627X00 | Diffuse non-Hodgkin's lymphoma, unspecified |
| 39870 | B172.00 | Malignant neoplasm of tail of pancreas |
| 39878 | B327300 | Malignant melanoma of popliteal fossa area |
| 39897 | B081.00 | Malignant neoplasm of pyriform sinus |
| 39899 | B542100 | Malignant neoplasm of craniopharyngeal duct |
| 39923 | B223100 | Malignant neoplasm of middle lobe of lung |
| 40014 | B310100 | Malignant neoplasm of soft tissue of face |
| 40292 | B053.00 | Malignant neoplasm of soft palate |
| 40437 | B50y.00 | Malignant neoplasm of other specified site of eye |
| 40557 | B01z.00 | Malignant neoplasm of tongue NOS |
| 40592 | Byu5.00 | [X]Malignant neoplasm of mesothelial and soft tissue |
| 40595 | Byu2000 | [X]Malignant neoplasm of bronchus or lung, unspecified |
| 40598 | Byu7.00 | [X]Malignant neoplasm of female genital organs |
| 40608 | ByuB.00 | [X]Malignant neoplasm of thyroid and other endocrine glands |
| 40671 | Byu8.00 | [X]Malignant neoplasm of male genital organs |
| 40740 | ByuD.00 | [X]Malignant neoplasms of lymphoid, haematopoietic and rela |
| 40749 | Byu3.00 | [X]Malignant neoplasm of bone and articular cartilage |
| 40810 | B171.00 | Malignant neoplasm of body of pancreas |
| 40814 | B307200 | Malignant neoplasm of tibia |
| 40966 | B306300 | Malignant neoplasm of sacral vertebra |
| 41011 | B3z..00 | Malig neop of bone, connective tissue, skin and breast NOS |
| 41144 | B582300 | Secondary malignant neoplasm of skin of trunk |
| 41215 | B111100 | Malignant neoplasm of pyloric canal of stomach |
| 41278 | B323000 | Malignant melanoma of external surface of cheek |
| 41362 | B101.00 | Malignant neoplasm of thoracic oesophagus |
| 41369 | B60..00 | Lymphosarcoma and reticulosarcoma |
| 41490 | B327700 | Malignant melanoma of foot |
| 41515 | ByuA100 | [X]Malignant neoplasm/central nervous system, unspecified |
| 41520 | B51z.00 | Malignant neoplasm of brain NOS |
| 41523 | B223000 | Malignant neoplasm of middle lobe bronchus |
| 41530 | B01y.00 | Malignant neoplasm of other sites of tongue |
| 41571 | B495.00 | Malignant neoplasm of bladder neck |
| 41691 | B562000 | Secondary and unspec malig neop coeliac lymph nodes |
| 41931 | B550100 | Malignant neoplasm of cheek NOS |
| 42012 | B494.00 | Malignant neoplasm of posterior wall of urinary bladder |
| 42023 | B497.00 | Malignant neoplasm of urachus |
| 42070 | B345.00 | Malignant neoplasm of lower-outer quadrant of female breast |
| 42153 | B32y.00 | Malignant melanoma of other specified skin site |
| 42193 | B115.00 | Malignant neoplasm of lesser curve of stomach unspecified |
| 42218 | B55y.00 | Malignant neoplasm of other specified sites |
| 42416 | B105.00 | Malignant neoplasm of lower third of oesophagus |
| 42426 | B511.00 | Malignant neoplasm of frontal lobe |
| 42460 | B543.00 | Malignant neoplasm of pineal gland |
| 42461 | B61zz00 | Hodgkin's disease NOS |
| 42539 | B670.00 | Acute erythraemia and erythroleukaemia |
| 42566 | B224z00 | Malignant neoplasm of lower lobe, bronchus or lung NOS |
| 42569 | B2zz.00 | Malignant neoplasm of respiratory tract NOS |
| 42579 | B62y300 | Malignant lymphoma NOS of intra-abdominal lymph nodes |
| 42714 | B327500 | Malignant melanoma of ankle |
| 42856 | B200z00 | Malignant neoplasm of nasal cavities NOS |
| 43111 | B213.00 | Malignant neoplasm of laryngeal cartilage |
| 43151 | Byu3300 | [X]Malignant neoplasm/bone+articular cartilage, unspecified |
| 43200 | B06z.00 | Malignant neoplasm of oropharynx NOS |
| 43390 | B12z.00 | Malignant neoplasm of small intestine NOS |
| 43392 | B483.00 | Malignant neoplasm of penis, part unspecified |
| 43400 | B03..00 | Malignant neoplasm of gum |
| 43415 | ByuD000 | [X]Other Hodgkin's disease |
| 43431 | B010.00 | Malignant neoplasm of base of tongue |
| 43435 | B41yz00 | Malignant neoplasm of other site of cervix NOS |
| 43450 | B63z.00 | Immunoproliferative neoplasm or myeloma NOS |
| 43463 | B325700 | Malignant melanoma of back |
| 43475 | B310.00 | Malig neop of connective and soft tissue head, face and neck |
| 43479 | B121.00 | Malignant neoplasm of jejunum |
| 43490 | Byu1100 | [X]Other specified carcinomas of liver |
| 43548 | B080.00 | Malignant neoplasm of postcricoid region |
| 43552 | B630.11 | Kahler's disease |
| 43572 | B114.00 | Malignant neoplasm of body of stomach |
| 43614 | B30X.00 | Malignant neoplasm/bones+articular cartilage/limb,unspfd |
| 43642 | B011.00 | Malignant neoplasm of dorsal surface of tongue |
| 43715 | B325600 | Malignant melanoma of umbilicus |
| 43761 | B451.00 | Malignant neoplasm of labia majora |
| 43781 | B011z00 | Malignant neoplasm of dorsum of tongue NOS |
| 43930 | B582000 | Secondary malignant neoplasm of skin of head |
| 43940 | B431.00 | Malignant neoplasm of isthmus of uterine body |
| 44089 | B517.00 | Malignant neoplasm of brain stem |
| 44108 | B18..00 | Malignant neoplasm of retroperitoneum and peritoneum |
| 44139 | B073.00 | Malignant neoplasm of anterior wall of nasopharynx |
| 44169 | B222z00 | Malignant neoplasm of upper lobe, bronchus or lung NOS |
| 44196 | B611.00 | Hodgkin's granuloma |
| 44267 | B623.00 | Malignant histiocytosis |
| 44318 | B62xX00 | Oth and unspecif peripheral & cutaneous T-cell lymphomas |
| 44356 | B2z..00 | Malig neop other/ill-defined sites resp/intrathoracic organs |
| 44399 | B150z00 | Primary malignant neoplasm of liver NOS |
| 44452 | B300C00 | Malignant neoplasm of vomer |
| 44529 | B575.00 | Secondary malignant neoplasm of large intestine and rectum |
| 44609 | B306000 | Malignant neoplasm of ilium |
| 44615 | B586.00 | Secondary malignant neoplasm of ovary |
| 44627 | B560800 | Secondary and unspec malig neop anterior cervical LN |
| 44805 | B312100 | Malig neop of connective and soft tissue thigh and upper leg |
| 44884 | B4Ay.00 | Malignant neoplasm of other urinary organs |
| 44931 | B562z00 | Secondary and unspec malig neop intra-abdominal LN NOS |
| 44996 | B491.00 | Malignant neoplasm of dome of urinary bladder |
| 45071 | B314.00 | Malignant neoplasm of connective and soft tissue of abdomen |
| 45139 | B323400 | Malignant melanoma of external surface of nose |
| 45154 | B516.00 | Malignant neoplasm of cerebellum |
| 45222 | B343.00 | Malignant neoplasm of lower-inner quadrant of female breast |
| 45260 | Byu9000 | [X]Malignant neoplasm of urinary organ, unspecified |
| 45262 | Byu8200 | [X]Malignant neoplasm of male genital organ, unspecified |
| 45264 | B620100 | Nodular lymphoma of lymph nodes of head, face and neck |
| 45267 | B55z.00 | Malignant neoplasm of other and ill defined site NOS |
| 45306 | B324100 | Malignant melanoma of neck |
| 45307 | B2...11 | Carcinoma of respiratory tract and intrathoracic organs |
| 45408 | B040.00 | Malignant neoplasm of anterior portion of floor of mouth |
| 45490 | B430z00 | Malignant neoplasm of corpus uteri NOS |
| 45667 | B501.00 | Malignant neoplasm of orbit |
| 45700 | By...00 | Neoplasms otherwise specified |
| 45755 | B326200 | Malignant melanoma of fore-arm |
| 45760 | B325z00 | Malignant melanoma of trunk, excluding scrotum, NOS |
| 45766 | Byu1200 | [X]Malignant neoplasm of intestinal tract, part unspecified |
| 45793 | B430300 | Malignant neoplasm of myometrium of corpus uteri |
| 45824 | B58y900 | Secondary malignant neoplasm of tongue |
| 45922 | B508.00 | Malignant neoplasm, overlapping lesion of eye and adnexa |
| 45986 | B041.00 | Malignant neoplasm of lateral portion of floor of mouth |
| 46042 | B630300 | Lambda light chain myeloma |
| 46114 | B0z..00 | Malig neop other/ill-defined sites lip, oral cavity, pharynx |
| 46153 | B443.00 | Malignant neoplasm of parametrium |
| 46159 | B142000 | Malignant neoplasm of cloacogenic zone |
| 46255 | B327.00 | Malignant melanoma of lower limb and hip |
| 46409 | B563300 | Secondary and unspec malig neop pectoral lymph nodes |
| 46548 | B071100 | Malignant neoplasm of pharyngeal tonsil |
| 46613 | B18y.00 | Malignant neoplasm of specified parts of peritoneum |
| 46728 | B064.00 | Malignant neoplasm of anterior epiglottis |
| 46789 | B515000 | Malignant neoplasm of choroid plexus |
| 46792 | B512.00 | Malignant neoplasm of temporal lobe |
| 46905 | B545200 | Malignant neoplasm of coccygeal body |
| 46939 | B302000 | Malignant neoplasm of cervical vertebra |
| 47094 | B323200 | Malignant melanoma of eyebrow |
| 47204 | B625z00 | Letterer-Siwe disease NOS |
| 47205 | B017.00 | Malignant overlapping lesion of tongue |
| 47252 | B323.00 | Malignant melanoma of other and unspecified parts of face |
| 47286 | B551.00 | Malignant neoplasm of thorax |
| 47366 | B565300 | Secondary and unspec malig neop sacral lymph nodes |
| 47556 | B512z00 | Malignant neoplasm of temporal lobe NOS |
| 47633 | ByuA300 | [X]Malig neopl, overlap lesion brain & other part of CNS |
| 47668 | B48y100 | Malignant neoplasm of tunica vaginalis |
| 47767 | B486.00 | Malignant neoplasm of scrotum |
| 47801 | B49y000 | Malignant neoplasm, overlapping lesion of bladder |
| 47810 | B59..00 | Malignant neoplasm of unspecified site |
| 47840 | B545100 | Malignant neoplasm of aortic body |
| 47862 | B213300 | Malignant neoplasm of thyroid cartilage |
| 47899 | B451000 | Malignant neoplasm of greater vestibular (Bartholin's) gland |
| 48073 | B510000 | Malignant neoplasm of basal ganglia |
| 48231 | B13y.00 | Malignant neoplasm of other specified sites of colon |
| 48237 | B111000 | Malignant neoplasm of prepylorus of stomach |
| 48517 | B310200 | Malignant neoplasm of soft tissue of neck |
| 48519 | B065.00 | Malignant neoplasm of junctional region of epiglottis |
| 48537 | B17y.00 | Malignant neoplasm of other specified sites of pancreas |
| 48743 | B482.00 | Malignant neoplasm of body of penis |
| 48809 | B35zz00 | Malignant neoplasm of male breast NOS |
| 48820 | B410.00 | Malignant neoplasm of endocervix |
| 48828 | B582500 | Secondary malignant neoplasm of skin of hip and leg |
| 49054 | B304000 | Malignant neoplasm of scapula |
| 49132 | B517100 | Malignant neoplasm of medulla oblongata |
| 49145 | B58y700 | Secondary malignant neoplasm of penis |
| 49148 | B347.00 | Malignant neoplasm, overlapping lesion of breast |
| 49214 | B560.00 | Secondary and unspec malig neop lymph nodes head/face/neck |
| 49262 | B627200 | Follicular non-Hodgkin's large cell lymphoma |
| 49292 | Byu1300 | [X]Malignant neoplsm/ill-defin sites within digestive system |
| 49301 | B6z..00 | Malignant neoplasm lymphatic or haematopoietic tissue NOS |
| 49360 | B031.00 | Malignant neoplasm of lower gum |
| 49400 | B430211 | Malignant neoplasm of endometrium |
| 49463 | B310400 | Malignant neoplasm of tarsus of eyelid |
| 49491 | B303100 | Malignant neoplasm of sternum |
| 49525 | B59zX00 | Kaposi's sarcoma, unspecified |
| 49605 | B615.00 | Hodgkin's disease, mixed cellularity |
| 49701 | B302z00 | Malignant neoplasm of vertebral column NOS |
| 49714 | B523.00 | Malignant neoplasm of spinal meninges |
| 49725 | B64y.00 | Other lymphoid leukaemia |
| 49758 | B0zy.00 | Malignant neoplasm of other sites lip, oral cavity, pharynx |
| 49814 | B325000 | Malignant melanoma of axilla |
| 49828 | B441.00 | Malignant neoplasm of fallopian tube |
| 49875 | B52X.00 | Malignant neoplasm of meninges, unspecified |
| 50035 | B545.00 | Malignant neoplasm of aortic body and other paraganglia |
| 50152 | B306500 | Malignant sacral teratoma |
| 50199 | B563.00 | Secondary and unspec malig neop axilla and upper limb LN |
| 50222 | B311000 | Malignant neoplasm of connective and soft tissue of shoulder |
| 50285 | B410z00 | Malignant neoplasm of endocervix NOS |
| 50289 | B241z00 | Malignant neoplasm of heart NOS |
| 50290 | B6z0.00 | Kaposi's sarcoma of lymph nodes |
| 50292 | Byu2500 | [X]Malignant neoplasm of mediastinum, part unspecified |
| 50296 | B000100 | Malignant neoplasm of upper lip, lipstick area |
| 50297 | B411.00 | Malignant neoplasm of exocervix |
| 50298 | B300500 | Malignant neoplasm of orbital bone |
| 50299 | B300900 | Malignant neoplasm of zygomatic bone |
| 50402 | B307100 | Malignant neoplasm of fibula |
| 50475 | B02z.00 | Malignant neoplasm of major salivary gland NOS |
| 50505 | B326000 | Malignant melanoma of shoulder |
| 50579 | B214.00 | Malignant neoplasm, overlapping lesion of larynx |
| 50668 | B627300 | Diffuse non-Hodgkin's small cell (diffuse) lymphoma |
| 50681 | B480.00 | Malignant neoplasm of prepuce (foreskin) |
| 50695 | B627500 | Diffuse non-Hodgkin mixed sml & lge cell (diffuse) lymphoma |
| 50696 | B62y100 | Malignant lymphoma NOS of lymph nodes of head, face and neck |
| 50777 | B524600 | Malignant neoplasm,overlap lesion periph nerve & auton ns |
| 50789 | B103.00 | Malignant neoplasm of upper third of oesophagus |
| 50858 | B674.00 | Acute panmyelosis |
| 50898 | B18y300 | Malignant neoplasm of omentum |
| 50904 | B563200 | Secondary and unspec malig neop infraclavicular lymph nodes |
| 50974 | B14z.00 | Malignant neoplasm rectum,rectosigmoid junction and anus NOS |
| 51115 | B522.00 | Malignant neoplasm of spinal cord |
| 51209 | B325800 | Malignant melanoma of chest wall |
| 51237 | B303z00 | Malignant neoplasm of rib, sternum and clavicle NOS |
| 51255 | B1zz.00 | Malignant neoplasm of digestive tract and peritoneum NOS |
| 51352 | B592.00 | Malignant neoplasms of independent (primary) multiple sites |
| 51551 | B571.00 | Secondary malignant neoplasm of mediastinum |
| 51690 | B117.00 | Malignant neoplasm, overlapping lesion of stomach |
| 51786 | B021.00 | Malignant neoplasm of submandibular gland |
| 51795 | B545000 | Malignant neoplasm of glomus jugulare |
| 51818 | B550300 | Malignant neoplasm of jaw NOS |
| 51873 | B327100 | Malignant melanoma of thigh |
| 51921 | B306200 | Malignant neoplasm of pubis |
| 51926 | B062000 | Malignant neoplasm of faucial pillar |
| 51965 | B315.00 | Malignant neoplasm of connective and soft tissue of pelvis |
| 52029 | ByuC800 | [X]Malignant neoplasm without specification of site |
| 52190 | B561900 | Secondary and unspec malig neop pulmonary lymph nodes |
| 52316 | B553.00 | Malignant neoplasm of pelvis |
| 52327 | B653000 | Chloroma |
| 52511 | B515.00 | Malignant neoplasm of cerebral ventricles |
| 52537 | B161100 | Malignant neoplasm of hepatic duct |
| 52570 | B487.00 | Malignant neoplasm, overlapping lesion of penis |
| 52594 | B4z..00 | Malignant neoplasm of genitourinary organ NOS |
| 52736 | B562.00 | Secondary and unspec malig neop intra-abdominal lymph nodes |
| 53103 | B410100 | Malignant neoplasm of endocervical gland |
| 53369 | B327900 | Malignant melanoma of great toe |
| 53397 | B61z.00 | Hodgkin's disease NOS |
| 53504 | B52W.00 | Malig neopl, overlap lesion brain & other part of CNS |
| 53528 | B581200 | Secondary malignant neoplasm of urethra |
| 53551 | B627600 | Diffuse non-Hodgkin's immunoblastic (diffuse) lymphoma |
| 53591 | B10y.00 | Malignant neoplasm of other specified part of oesophagus |
| 53594 | B300000 | Malignant neoplasm of ethmoid bone |
| 53599 | B300100 | Malignant neoplasm of frontal bone |
| 53629 | B325200 | Malignant melanoma of buttock |
| 53884 | B060z00 | Malignant neoplasm tonsil NOS |
| 53910 | B453.00 | Malignant neoplasm of clitoris |
| 53989 | B311.00 | Malig neop connective and soft tissue upper limb/shoulder |
| 54083 | B625800 | Letterer-Siwe disease of lymph nodes of multiple sites |
| 54103 | B16..00 | Malignant neoplasm gallbladder and extrahepatic bile ducts |
| 54120 | B584.00 | Secondary malignant neoplasm of other part of nervous system |
| 54133 | B510z00 | Malignant neoplasm of cerebrum NOS |
| 54134 | B223z00 | Malignant neoplasm of middle lobe, bronchus or lung NOS |
| 54171 | B104.00 | Malignant neoplasm of middle third of oesophagus |
| 54184 | B4A1z00 | Malignant neoplasm of renal pelvis NOS |
| 54186 | B313100 | Malignant neoplasm of diaphragm |
| 54202 | B35z.00 | Malignant neoplasm of other site of male breast |
| 54222 | B312400 | Malignant neoplasm of connective and soft tissue of foot |
| 54253 | ByuC700 | [X]Secondary malignant neoplasm of other specified sites |
| 54267 | B59z.00 | Malignant neoplasm of unspecified site NOS |
| 54278 | B564000 | Secondary and unspec malig neop superficial inguinal LN |
| 54305 | B327200 | Malignant melanoma of knee |
| 54493 | B303500 | Malignant neoplasm of xiphoid process |
| 54494 | B350.00 | Malignant neoplasm of nipple and areola of male breast |
| 54613 | B201200 | Malignant neoplasm of tympanic antrum |
| 54631 | B306.00 | Malignant neoplasm of pelvic bones, sacrum and coccyx |
| 54632 | B321.00 | Malignant melanoma of eyelid including canthus |
| 54636 | B203.00 | Malignant neoplasm of ethmoid sinus |
| 54679 | B594.00 | Secondary malignant neoplasm of unknown site |
| 54685 | B326100 | Malignant melanoma of upper arm |
| 54691 | B302200 | Malignant neoplasm of lumbar vertebra |
| 54747 | B300600 | Malignant neoplasm of parietal bone |
| 54793 | B682.00 | Subacute leukaemia NOS |
| 54956 | B50z.00 | Malignant neoplasm of eye NOS |
| 54965 | B312200 | Malig neop connective and soft tissue of popliteal space |
| 55015 | B05z.00 | Malignant neoplasm of mouth NOS |
| 55019 | B11y.00 | Malignant neoplasm of other specified site of stomach |
| 55066 | B062.00 | Malignant neoplasm of tonsillar pillar |
| 55090 | B58y100 | Secondary malignant neoplasm of uterus |
| 55096 | B582z00 | Secondary malignant neoplasm of skin NOS |
| 55098 | B550000 | Malignant neoplasm of head NOS |
| 55101 | B553z00 | Malignant neoplasm of pelvis NOS |
| 55246 | B20z.00 | Malignant neoplasm of accessory sinus NOS |
| 55292 | B326z00 | Malignant melanoma of upper limb or shoulder NOS |
| 55303 | B614100 | Hodgkin's nodular sclerosis of head, face and neck |
| 55374 | B215.00 | Malignant neoplasm of epiglottis NOS |
| 55434 | B116.00 | Malignant neoplasm of greater curve of stomach unspecified |
| 55463 | B561400 | Secondary and unspec malig neop post mediastinal lymph nodes |
| 55588 | Byu7300 | [X]Malignant neoplasm of female genital organ, unspecified |
| 55595 | B300700 | Malignant neoplasm of sphenoid bone |
| 55630 | B07y.00 | Malignant neoplasm of other specified site of nasopharynx |
| 55659 | B14y.00 | Malig neop other site rectum, rectosigmoid junction and anus |
| 55881 | B324000 | Malignant melanoma of scalp |
| 55946 | B574000 | Secondary malignant neoplasm of duodenum |
| 55953 | B300400 | Malignant neoplasm of occipital bone |
| 56345 | B57y.00 | Secondary malignant neoplasm of other digestive organ |
| 56355 | B066.00 | Malignant neoplasm of lateral wall of oropharynx |
| 56490 | B52z.00 | Malignant neoplasm of nervous system NOS |
| 56513 | B307000 | Malignant neoplasm of femur |
| 56709 | B04y.00 | Malignant neoplasm of other sites of floor of mouth |
| 56715 | B34y.00 | Malignant neoplasm of other site of female breast |
| 56718 | B500z00 | Malignant neoplasm of eyeball NOS |
| 56918 | B1zy.00 | Malignant neoplasm other spec digestive tract and peritoneum |
| 56925 | Byu4000 | [X]Malignant melanoma of other+unspecified parts of face |
| 57047 | B544.00 | Malignant neoplasm of carotid body |
| 57191 | Byu8000 | [X]Malignant neoplasm/other specified male genital organs |
| 57225 | B614000 | Hodgkin's disease, nodular sclerosis of unspecified site |
| 57235 | B410000 | Malignant neoplasm of endocervical canal |
| 57248 | B082.00 | Malignant neoplasm aryepiglottic fold, hypopharyngeal aspect |
| 57260 | B322.00 | Malignant melanoma of ear and external auricular canal |
| 57427 | B62y000 | Malignant lymphoma NOS of unspecified site |
| 57471 | B316.00 | Malig neop of connective and soft tissue trunk unspecified |
| 57481 | ByuC300 | [X]Secondary malignant neoplasm/oth+unspc respiratory organs |
| 57482 | B311200 | Malignant neoplasm of connective and soft tissue of fore-arm |
| 57671 | B672.00 | Megakaryocytic leukaemia |
| 57719 | B41y100 | Malignant neoplasm of squamocolumnar junction of cervix |
| 57737 | B62x100 | Lymphoepithelioid lymphoma |
| 57756 | Byu7100 | [X]Malignant neoplasm/other specified female genital organs |
| 57854 | B553000 | Malignant neoplasm of inguinal region NOS |
| 57988 | B305000 | Malignant neoplasm of carpal bone - scaphoid |
| 58061 | B452.00 | Malignant neoplasm of labia minora |
| 58082 | B620800 | Nodular lymphoma of lymph nodes of multiple sites |
| 58088 | B151400 | Malignant neoplasm of intrahepatic gall duct |
| 58094 | B412.00 | Malignant neoplasm, overlapping lesion of cervix uteri |
| 58121 | B014.00 | Malignant neoplasm of anterior 2/3 of tongue unspecified |
| 58684 | B615200 | Hodgkin's mixed cellularity of intrathoracic lymph nodes |
| 58692 | B561500 | Secondary and unspec malig neop paratracheal lymph nodes |
| 58836 | B315z00 | Malig neop of connective and soft tissue of pelvis NOS |
| 58871 | B623z00 | Malignant histiocytosis NOS |
| 58903 | B550z00 | Malignant neoplasm of head, neck and face NOS |
| 58949 | B308D00 | Malignant neoplasm of phalanges of foot |
| 58958 | B323500 | Malignant melanoma of temple |
| 58962 | B62x500 | Malignant immunoproliferative small intestinal disease |
| 58973 | Byu0.00 | [X]Malignant neoplasm of lip, oral cavity and pharynx |
| 59004 | B072.00 | Malignant neoplasm of lateral wall of nasopharynx |
| 59036 | B300.00 | Malignant neoplasm of bones of skull and face |
| 59041 | B500000 | Malignant neoplasm of ciliary body |
| 59061 | B322000 | Malignant melanoma of auricle (ear) |
| 59092 | B111z00 | Malignant neoplasm of pylorus of stomach NOS |
| 59097 | B431000 | Malignant neoplasm of lower uterine segment |
| 59115 | B602100 | Burkitt's lymphoma of lymph nodes of head, face and neck |
| 59152 | B315200 | Malignant neoplasm of connective and soft tissue of perineum |
| 59170 | B51y000 | Malignant neoplasm of corpus callosum |
| 59223 | B306100 | Malignant neoplasm of ischium |
| 59286 | B4Ay000 | Malignant neoplasm of overlapping lesion of urinary organs |
| 59362 | B451z00 | Malignant neoplasm of labia majora NOS |
| 59375 | B583z00 | Secondary malignant neoplasm of brain or spinal cord NOS |
| 59381 | B500100 | Malignant neoplasm of iris |
| 59382 | B310000 | Malignant neoplasm of soft tissue of head |
| 59388 | B18y100 | Malignant neoplasm of mesocaecum |
| 59520 | B300200 | Malignant neoplasm of malar bone |
| 59718 | B542z00 | Malig neop pituitary gland or craniopharyngeal duct NOS |
| 59755 | B61z200 | Hodgkin's disease NOS of intrathoracic lymph nodes |
| 59778 | B61z100 | Hodgkin's disease NOS of lymph nodes of head, face and neck |
| 59823 | B542.00 | Malignant neoplasm pituitary gland and craniopharyngeal duct |
| 59831 | B340z00 | Malignant neoplasm of nipple or areola of female breast NOS |
| 60035 | B310300 | Malignant neoplasm of cartilage of ear |
| 60052 | B55yz00 | Malignant neoplasm of specified site NOS |
| 60053 | Byu..00 | [X]Additional neoplasm classification terms |
| 60092 | B62y700 | Malignant lymphoma NOS of spleen |
| 60134 | B581000 | Secondary malignant neoplasm of ureter |
| 60162 | Byu5A00 | [X]Malignant neoplasm overlapping lesion of skin |
| 60242 | B600000 | Reticulosarcoma of unspecified site |
| 60247 | B314z00 | Malig neop of connective and soft tissue of abdomen NOS |
| 60312 | B16y.00 | Malignant neoplasm other gallbladder/extrahepatic bile duct |
| 60335 | B58y400 | Secondary malignant neoplasm of vulva |
| 60403 | B303300 | Malignant neoplasm of costal cartilage |
| 60772 | B450z00 | Malignant neoplasm of vagina NOS |
| 61064 | B24X.00 | Malignant neoplasm of mediastinum, part unspecified |
| 61149 | B614300 | Hodgkin's nodular sclerosis of intra-abdominal lymph nodes |
| 61246 | B327600 | Malignant melanoma of heel |
| 61289 | B564100 | Secondary and unspec malig neop deep inguinal lymph nodes |
| 61390 | B540000 | Malignant neoplasm of adrenal cortex |
| 61399 | B510100 | Malignant neoplasm of cerebral cortex |
| 61500 | B690.00 | Acute myelomonocytic leukaemia |
| 61510 | B062200 | Malignant neoplasm of palatoglossal arch |
| 61555 | B180z00 | Malignant neoplasm of retroperitoneum NOS |
| 61643 | B151z00 | Malignant neoplasm of intrahepatic bile ducts NOS |
| 61662 | B61z000 | Hodgkin's disease NOS, unspecified site |
| 61677 | B562200 | Secondary and unspec malig neop inferior mesenteric LN |
| 61692 | B004.00 | Malignant neoplasm of lip unspecified, inner aspect |
| 61693 | ByuD600 | [X]Other myeloid leukaemia |
| 61695 | B100.00 | Malignant neoplasm of cervical oesophagus |
| 61716 | B524100 | Malignant neoplasm of peripheral nerve,upp limb,incl should |
| 61741 | B304200 | Malignant neoplasm of humerus |
| 62104 | B300800 | Malignant neoplasm of temporal bone |
| 62124 | B561800 | Secondary and unspec malig neop bronchopulmonary lymph nodes |
| 62126 | B510500 | Malignant neoplasm of thalamus |
| 62182 | B200300 | Malignant neoplasm of vestibule of nose |
| 62380 | B601200 | Lymphosarcoma of intrathoracic lymph nodes |
| 62437 | B62x400 | Malignant reticulosis |
| 62475 | B326300 | Malignant melanoma of hand |
| 62556 | B24..00 | Malignant neoplasm of thymus, heart and mediastinum |
| 62584 | B573.00 | Secondary malignant neoplasm of other respiratory organs |
| 62630 | B307z00 | Malignant neoplasm of long bones of leg NOS |
| 62761 | B200200 | Malignant neoplasm of septum of nose |
| 62828 | B581z00 | Secondary malignant neoplasm of other urinary organ NOS |
| 62840 | B013.00 | Malignant neoplasm of ventral surface of tongue |
| 62909 | B575100 | Secondary malignant neoplasm of rectum |
| 63054 | B614z00 | Hodgkin's disease, nodular sclerosis NOS |
| 63104 | B501z00 | Malignant neoplasm of orbit NOS |
| 63105 | B62y500 | Malignant lymphoma NOS of lymph node inguinal region and leg |
| 63224 | B48z.00 | Malignant neoplasm of penis and other male genital organ NOS |
| 63300 | Byu3200 | [X]Malignant neoplasm/overlap lesion/bone+articulr cartilage |
| 63331 | B485.00 | Malignant neoplasm of spermatic cord |
| 63375 | ByuDE00 | [X]Unspecified B-cell non-Hodgkin's lymphoma |
| 63430 | B241000 | Malignant neoplasm of endocardium |
| 63460 | B213000 | Malignant neoplasm of arytenoid cartilage |
| 63470 | B102.00 | Malignant neoplasm of abdominal oesophagus |
| 63475 | B652.00 | Subacute myeloid leukaemia |
| 63568 | B524000 | Malignant neoplasm of peripheral nerves of head, face & neck |
| 63598 | ByuE.00 | [X]Malignant neoplasms/independent (primary) multiple sites |
| 63625 | B616400 | Hodgkin's lymphocytic depletion lymph nodes axilla and arm |
| 63653 | B671.11 | Heilmeyer - Schoner disease |
| 63657 | B503.00 | Malignant neoplasm of conjunctiva |
| 63695 | B524300 | Malignant neoplasm of peripheral nerve of thorax |
| 63723 | B601z00 | Lymphosarcoma NOS |
| 63896 | B582400 | Secondary malignant neoplasm of skin of shoulder and arm |
| 63915 | B564.00 | Secondary and unspec malig neop inguinal and lower limb LN |
| 63925 | ByuA200 | [X]Malignant neoplasm of meninges, unspecified |
| 63979 | B013100 | Malignant neoplasm of frenulum linguae |
| 63988 | B311500 | Malignant neoplasm of connective and soft tissue of thumb |
| 63995 | B123.00 | Malignant neoplasm of Meckel's diverticulum |
| 63997 | B326500 | Malignant melanoma of thumb |
| 64036 | B612.00 | Hodgkin's sarcoma |
| 64106 | B18yz00 | Malignant neoplasm of specified parts of peritoneum NOS |
| 64116 | B561.00 | Secondary and unspec malig neop intrathoracic lymph nodes |
| 64195 | B54z.00 | Malig neop of endocrine gland or related structure NOS |
| 64309 | ByuB100 | [X]Malignant neoplasm of endocrine gland, unspecified |
| 64327 | B327z00 | Malignant melanoma of lower limb or hip NOS |
| 64336 | ByuD300 | [X]Other specified types of non-Hodgkin's lymphoma |
| 64345 | B311100 | Malignant neoplasm of connective and soft tissue, upper arm |
| 64427 | B62z100 | Unspec malig neop lymphoid/histiocytic lymph node head/neck |
| 64462 | B083.00 | Malignant neoplasm of posterior pharynx |
| 64497 | Byu7000 | [X]Malignant neoplasm of uterine adnexa, unspecified |
| 64515 | ByuDC00 | [X]Diffuse non-Hodgkin's lymphoma, unspecified |
| 64516 | B18y400 | Malignant neoplasm of parietal peritoneum |
| 64557 | B517000 | Malignant neoplasm of cerebral peduncle |
| 64567 | B63y.00 | Other immunoproliferative neoplasms |
| 64602 | B470.00 | Malignant neoplasm of undescended testis |
| 64670 | B601300 | Lymphosarcoma of intra-abdominal lymph nodes |
| 64680 | B574.00 | Secondary malignant neoplasm of small intestine and duodenum |
| 64686 | B340100 | Malignant neoplasm of areola of female breast |
| 64810 | B551z00 | Malignant neoplasm of thorax NOS |
| 64817 | B502.00 | Malignant neoplasm of lacrimal gland |
| 64848 | B304400 | Malignant neoplasm of ulna |
| 64897 | ByuE000 | [X]Malignant neoplasms/independent(primary)multiple sites |
| 64918 | B560000 | Secondary and unspec malig neop of superficial parotid LN |
| 64971 | B520000 | Malignant neoplasm of olfactory bulb |
| 65106 | B44z.00 | Malignant neoplasm of uterine adnexa NOS |
| 65122 | B624000 | Leukaemic reticuloendotheliosis of unspecified sites |
| 65123 | B624300 | Leukaemic reticuloend of intra-abdominal lymph nodes |
| 65124 | B151000 | Malignant neoplasm of interlobular bile ducts |
| 65159 | B180100 | Malignant neoplasm of perinephric tissue |
| 65164 | B326.00 | Malignant melanoma of upper limb and shoulder |
| 65165 | ByuD900 | [X]Other leukaemia of unspecified cell type |
| 65180 | B627800 | Diffuse non-Hodgkin's lymphoma undifferentiated (diffuse) |
| 65215 | B205.00 | Malignant neoplasm of sphenoidal sinus |
| 65233 | B31y.00 | Malig neop connective and soft tissue other specified site |
| 65241 | B51y200 | Malignant neoplasm, overlapping lesion of brain |
| 65253 | B560300 | Secondary and unspec malignant neoplasm occipital lymph node |
| 65312 | B11y000 | Malignant neoplasm of anterior wall of stomach NEC |
| 65357 | B507100 | Malignant neoplasm of nasolacrimal duct |
| 65372 | B11yz00 | Malignant neoplasm of other specified site of stomach NOS |
| 65434 | B62z.00 | Malignant neoplasms of lymphoid and histiocytic tissue NOS |
| 65458 | B52..00 | Malig neop of other and unspecified parts of nervous system |
| 65460 | B1z1.00 | Malignant neoplasm of spleen NEC |
| 65466 | B592X00 | Kaposi's sarcoma of multiple organs |
| 65483 | B614400 | Hodgkin's nodular sclerosis of lymph nodes of axilla and arm |
| 65489 | B610.00 | Hodgkin's paragranuloma |
| 65490 | B58y411 | Secondary cancer of the vulva |
| 65599 | B520200 | Malignant neoplasm of acoustic nerve |
| 65605 | B241200 | Malignant neoplasm of myocardium |
| 65625 | B324.00 | Malignant melanoma of scalp and neck |
| 65642 | B623300 | Malignant histiocytosis of intra-abdominal lymph nodes |
| 65701 | B620z00 | Nodular lymphoma NOS |
| 65721 | B673.00 | Mast cell leukaemia |
| 65777 | B672.11 | Thrombocytic leukaemia |
| 65793 | B2z0.00 | Malig neop of upper respiratory tract, part unspecified |
| 65880 | B304z00 | Malig neop of scapula and long bones of upper arm NOS |
| 66083 | B57z.00 | Secondary malig neop of respiratory or digestive system NOS |
| 66088 | B312.00 | Malig neop of connective and soft tissue of hip and leg |
| 66089 | B65yz00 | Other myeloid leukaemia NOS |
| 66163 | ByuC200 | [X]2ndry+unspcf malignant neoplasm lymph nodes/multi regions |
| 66166 | B124.00 | Malignant neoplasm, overlapping lesion of small intestine |
| 66270 | B000000 | Malignant neoplasm of upper lip, external |
| 66327 | B620000 | Nodular lymphoma of unspecified site |
| 66384 | B001000 | Malignant neoplasm of lower lip, external |
| 66422 | B074.00 | Malignant neoplasm, overlapping lesion of nasopharynx |
| 66444 | Byu2100 | [X]Malignant neoplasm/overlap lesion/heart,mediastinm+pleura |
| 66488 | B314000 | Malig neop of connective and soft tissue of abdominal wall |
| 66639 | B303200 | Malignant neoplasm of clavicle |
| 66646 | B26..00 | Malignant neoplasm, overlap lesion of resp & intrathor orgs |
| 66750 | B24z.00 | Malignant neoplasm of heart, thymus and mediastinum NOS |
| 66775 | B560100 | Secondary and unspec malignant neoplasm mastoid lymph nodes |
| 66908 | B306400 | Malignant neoplasm of coccygeal vertebra |
| 67029 | ByuD500 | [X]Other lymphoid leukaemia |
| 67034 | Byu5000 | [X]Mesothelioma of other sites |
| 67107 | B230.00 | Malignant neoplasm of parietal pleura |
| 67129 | B560z00 | Secondary unspec malig neop lymph nodes head/face/neck NOS |
| 67211 | B523z00 | Malignant neoplasm of spinal meninges NOS |
| 67217 | B55y100 | Malignant neoplasm of trunk NOS |
| 67236 | B512000 | Malignant neoplasm of hippocampus |
| 67323 | B06y.00 | Malignant neoplasm of oropharynx, other specified sites |
| 67324 | B315100 | Malig neop of connective and soft tissue of inguinal region |
| 67396 | B576.00 | Secondary malig neop of retroperitoneum and peritoneum |
| 67446 | B001.00 | Malignant neoplasm of lower lip, vermilion border |
| 67451 | B30W.00 | Malignant neoplasm/overlap lesion/bone+articulr cartilage |
| 67497 | B106.00 | Malignant neoplasm, overlapping lesion of oesophagus |
| 67504 | B003000 | Malignant neoplasm of lower lip, buccal aspect |
| 67506 | B614200 | Hodgkin's nodular sclerosis of intrathoracic lymph nodes |
| 67518 | ByuD100 | [X]Other types of follicular non-Hodgkin's lymphoma |
| 67700 | B66..12 | Monoblastic leukaemia |
| 67703 | B616.00 | Hodgkin's disease, lymphocytic depletion |
| 67763 | B303400 | Malignant neoplasm of costo-vertebral joint |
| 67797 | B561600 | Secondary and unspec malig neop superfic tracheobronchial LN |
| 67806 | B323z00 | Malignant melanoma of face NOS |
| 67884 | B350100 | Malignant neoplasm of areola of male breast |
| 67949 | B48y.00 | Malignant neoplasm of other male genital organ |
| 68027 | ByuA000 | [X]Malignant neoplasm/other and unspecified cranial nerves |
| 68039 | B612400 | Hodgkin's sarcoma of lymph nodes of axilla and upper limb |
| 68055 | B307.00 | Malignant neoplasm of long bones of leg |
| 68133 | B323300 | Malignant melanoma of forehead |
| 68155 | B430100 | Malignant neoplasm of fundus of corpus uteri |
| 68161 | B48y000 | Malignant neoplasm of seminal vesicle |
| 68236 | B550.00 | Malignant neoplasm of head, neck and face |
| 68330 | B613100 | Hodgkin's, lymphocytic-histiocytic pred of head, face, neck |
| 68332 | ByuC600 | [X]2ndry malignant neoplasm/oth+unspec parts/nervous system |
| 68399 | B004200 | Malignant neoplasm of lip unspecified, mucosa |
| 68410 | B150200 | Primary angiosarcoma of liver |
| 68480 | B350000 | Malignant neoplasm of nipple of male breast |
| 68611 | B560900 | Secondary and unspec malig neop deep cervical LN |
| 68641 | B517z00 | Malignant neoplasm of brain stem NOS |
| 68787 | B55y000 | Malignant neoplasm of back NOS |
| 68824 | B48y200 | Malignant neoplasm, overlapping lesion male genital orgs |
| 69104 | B305100 | Malignant neoplasm of carpal bone - lunate |
| 69132 | B562400 | Secondary and unspec malig neop external iliac lymph nodes |
| 69146 | B300z00 | Malignant neoplasm of bones of skull and face NOS |
| 69392 | B561700 | Secondary and unspec malig neop inferior tracheobronchial LN |
| 69497 | B623000 | Malignant histiocytosis of unspecified site |
| 69671 | B010.11 | Malignant neoplasm of posterior third of tongue |
| 69761 | B00zz00 | Malignant neoplasm of lip, vermilion border NOS |
| 69821 | B18y600 | Malignant neoplasm of the pouch of Douglas |
| 69927 | B308800 | Malignant neoplasm of first metatarsal bone |
| 69951 | B055100 | Malignant neoplasm of roof of mouth |
| 70026 | B574z00 | Secondary malig neop of small intestine or duodenum NOS |
| 70104 | B521z00 | Malignant neoplasm of cerebral meninges NOS |
| 70126 | B520100 | Malignant neoplasm of optic nerve |
| 70374 | B600300 | Reticulosarcoma of intra-abdominal lymph nodes |
| 70463 | B315000 | Malignant neoplasm of connective and soft tissue of buttock |
| 70509 | B627D00 | Diffuse non-Hodgkin's centroblastic lymphoma |
| 70637 | B320.00 | Malignant melanoma of lip |
| 70696 | B02y.00 | Malignant neoplasm of other major salivary glands |
| 70716 | B62zz11 | Immunoproliferative neoplasm |
| 70724 | B653.00 | Myeloid sarcoma |
| 70729 | B431z00 | Malignant neoplasm of isthmus of uterine body NOS |
| 70736 | B58y300 | Secondary malignant neoplasm of vagina |
| 70747 | B564z00 | Secondary and unspec malig neop of inguinal and leg LN NOS |
| 70819 | B055.00 | Malignant neoplasm of palate unspecified |
| 70824 | B540z00 | Malignant neoplasm of adrenal gland NOS |
| 70842 | B627100 | Follicular non-Hodg mixed sml cleavd & lge cell lymphoma |
| 70928 | B022.00 | Malignant neoplasm of sublingual gland |
| 70942 | B510400 | Malignant neoplasm of hypothalamus |
| 71031 | B600100 | Reticulosarcoma of lymph nodes of head, face and neck |
| 71136 | B323100 | Malignant melanoma of chin |
| 71139 | B51y.00 | Malignant neoplasm of other parts of brain |
| 71142 | B613000 | Hodgkin's, lymphocytic-histiocytic predominance unspec site |
| 71147 | B003.00 | Malignant neoplasm of lower lip, inner aspect |
| 71204 | B200000 | Malignant neoplasm of cartilage of nose |
| 71238 | B601100 | Lymphosarcoma of lymph nodes of head, face and neck |
| 71262 | B62y600 | Malignant lymphoma NOS of intrapelvic lymph nodes |
| 71304 | B602z00 | Burkitt's lymphoma NOS |
| 71584 | B507.00 | Malignant neoplasm of lacrimal duct |
| 71609 | B62z500 | Unspec malig neop lymphoid/histiocytic nodes inguinal/leg |
| 71625 | B601000 | Lymphosarcoma of unspecified site |
| 71810 | B304.00 | Malignant neoplasm of scapula and long bones of upper arm |
| 71946 | B201300 | Malignant neoplasm of mastoid air cells |
| 72127 | B484.00 | Malignant neoplasm of epididymis |
| 72174 | B4A4.00 | Malignant neoplasm of paraurethral glands |
| 72197 | B67y000 | Lymphosarcoma cell leukaemia |
| 72212 | B308200 | Malignant neoplasm of calcaneum |
| 72224 | B1z1100 | Fibrosarcoma of spleen |
| 72445 | B161000 | Malignant neoplasm of cystic duct |
| 72464 | B305.12 | Malignant neoplasm of metacarpal bones |
| 72500 | ByuDB00 | [X]Mal neoplasm/lymphoid,haematopoietic+related tissu,unspcf |
| 72522 | B313200 | Malignant neoplasm of great vessels |
| 72713 | B562100 | Secondary and unspec malig neop superficial mesenteric LN |
| 72714 | B621500 | Mycosis fungoides of lymph nodes of inguinal region and leg |
| 72723 | B430000 | Malignant neoplasm of cornu of corpus uteri |
| 72725 | B62y200 | Malignant lymphoma NOS of intrathoracic lymph nodes |
| 72774 | B642.00 | Subacute lymphoid leukaemia |
| 72803 | B565z00 | Secondary and unspec malig neop intrapelvic LN NOS |
| 73213 | B581.00 | Secondary malignant neoplasm of other urinary organs |
| 73296 | Byu3100 | [X]Malignant neoplasm/bones+articular cartilage/limb,unspfd |
| 73439 | B064z00 | Malignant neoplasm of anterior epiglottis NOS |
| 73510 | B550500 | Malignant neoplasm of supraclavicular fossa NOS |
| 73530 | B305.00 | Malignant neoplasm of hand bones |
| 73532 | B613300 | Hodgkin's, lymphocytic-histiocytic pred intra-abdominal node |
| 73536 | B327000 | Malignant melanoma of hip |
| 73537 | B201z00 | Malig neop auditory tube, middle ear, mastoid air cells NOS |
| 73538 | B563z00 | Secondary and unspec malig neop axilla and upper limb LN NOS |
| 73556 | B305z00 | Malignant neoplasm of hand bones NOS |
| 73614 | B004000 | Malignant neoplasm of lip unspecified, buccal aspect |
| 73616 | B58y200 | Secondary malignant neoplasm of cervix uteri |
| 73718 | B310z00 | Malig neop connective and soft tissue head, face, neck NOS |
| 73744 | B322z00 | Malignant melanoma of ear and external auricular canal NOS |
| 73777 | B624z00 | Leukaemic reticuloendotheliosis NOS |
| 73962 | B000.00 | Malignant neoplasm of upper lip, vermilion border |
| 73988 | B524500 | Malignant neoplasm of peripheral nerve of pelvis |
| 73992 | B504.00 | Malignant neoplasm of cornea |
| 74896 | B161z00 | Malignant neoplasm of extrahepatic bile ducts NOS |
| 84368 | B565000 | Secondary and unspec malig neop internal iliac lymph nodes |
| 86046 | B524400 | Malignant neoplasm of peripheral nerve of abdomen |
| 86812 | B305D00 | Malignant neoplasm of phalanges of hand |
| 86996 | B501000 | Malignant neoplasm of connective tissue of orbit |
| 86997 | Byu2400 | [X]Malignant neoplasm/ill-defined sites within resp system |
| 87113 | B54X.00 | Malignant neoplasm-pluriglandular involvement,unspecified |
| 87335 | B624.12 | Hairy cell leukaemia |
| 88022 | ByuC400 | [X]Secondary malignant neoplasm/oth+unspcfd digestive organs |
| 88144 | B52y.00 | Malignant neoplasm of other specified part of nervous system |
| 88362 | B08y.00 | Malignant neoplasm of other specified hypopharyngeal site |
| 89258 | B524200 | Malignant neoplasm of peripheral nerve of low limb, incl hip |
| 89329 | ByuD800 | [X]Other specified leukaemias |
| 89593 | B151200 | Malignant neoplasm of intrahepatic biliary passages |
| 89657 | B626z00 | Malignant mast cell tumour NOS |
| 89762 | ByuD700 | [X]Other monocytic leukaemia |
| 89909 | B003200 | Malignant neoplasm of lower lip, mucosa |
| 89916 | B553100 | Malignant neoplasm of presacral region |
| 90124 | B067.00 | Malignant neoplasm of posterior wall of oropharynx |
| 90201 | B62x000 | T-zone lymphoma |
| 90290 | B18y700 | Malignant neoplasm of mesentery |
| 90546 | B312z00 | Malig neop connective and soft tissue hip and leg NOS |
| 90610 | B002300 | Malignant neoplasm of upper lip, oral aspect |
| 90659 | B54y.00 | Malignant neoplasm of other specified endocrine gland |
| 91035 | B010z00 | Malignant neoplasm of fixed part of tongue NOS |
| 91037 | B06yz00 | Malignant neoplasm of other specified site of oropharynx NOS |
| 91240 | B517300 | Malignant neoplasm of pons |
| 91457 | Byu5900 | [X]Malignant neoplasm/connective + soft tissue,unspecified |
| 91509 | B471z00 | Malignant neoplasm of descended testis NOS |
| 91586 | B311400 | Malignant neoplasm of connective and soft tissue of finger |
| 91674 | B621300 | Mycosis fungoides of intra-abdominal lymph nodes |
| 91843 | B003100 | Malignant neoplasm of lower lip, frenulum |
| 91895 | B064100 | Malignant neoplasm of glossoepiglottic fold |
| 91896 | Byu5800 | [X]Mal neoplasm/connective+soft tissue of trunk,unspecified |
| 91900 | B61z400 | Hodgkin's disease NOS of lymph nodes of axilla and arm |
| 92068 | B620300 | Nodular lymphoma of intra-abdominal lymph nodes |
| 92245 | B613200 | Hodgkin's, lymphocytic-histiocytic pred intrathoracic nodes |
| 92329 | B48yz00 | Malignant neoplasm of other male genital organ NOS |
| 92371 | B304300 | Malignant neoplasm of radius |
| 92380 | B602500 | Burkitt's lymphoma of lymph nodes of inguinal region and leg |
| 92382 | B308B00 | Malignant neoplasm of fourth metatarsal bone |
| 92703 | B560400 | Secondary and unspec malig neop deep parotid lymph nodes |
| 92720 | B243.00 | Malignant neoplasm of posterior mediastinum |
| 93218 | B03z.00 | Malignant neoplasm of gum NOS |
| 93342 | B66z.00 | Monocytic leukaemia NOS |
| 93384 | B62z200 | Unspec malig neop lymphoid/histiocytic of intrathoracic node |
| 93478 | B138.00 | Malignant neoplasm, overlapping lesion of colon |
| 93537 | B517200 | Malignant neoplasm of midbrain |
| 93665 | Byu5300 | [X]Kaposi's sarcoma, unspecified |
| 93716 | B561z00 | Secondary and unspec malig neop intrathoracic LN NOS |
| 93762 | B42..00 | Malignant neoplasm of placenta |
| 93778 | B1z1z00 | Malignant neoplasm of spleen NOS |
| 93842 | B062300 | Malignant neoplasm of palatopharyngeal arch |
| 93951 | B613500 | Hodgkin's, lymphocytic-histiocytic pred inguinal and leg |
| 94005 | B615z00 | Hodgkin's disease, mixed cellularity NOS |
| 94174 | B67y.00 | Other and unspecified leukaemia |
| 94220 | B540100 | Malignant neoplasm of adrenal medulla |
| 94251 | B00z100 | Malignant neoplasm of lip, unspecified, lipstick area |
| 94272 | B314100 | Malig neoplasm of connective and soft tissues of lumb spine |
| 94278 | B110111 | Malignant neoplasm of gastro-oesophageal junction |
| 94279 | B61z700 | Hodgkin's disease NOS of spleen |
| 94355 | B55y200 | Malignant neoplasm of flank NOS |
| 94390 | B070.00 | Malignant neoplasm of roof of nasopharynx |
| 94407 | B615100 | Hodgkin's mixed cellularity of lymph nodes head, face, neck |
| 94415 | B623100 | Malignant histiocytosis of lymph nodes head, face and neck |
| 94427 | B305C00 | Malignant neoplasm of fifth metacarpal bone |
| 94441 | B003300 | Malignant neoplasm of lower lip, oral aspect |
| 94776 | B1z2.00 | Malignant neoplasm, overlapping lesion of digestive system |
| 94975 | B241300 | Malignant neoplasm of pericardium |
| 94995 | B620500 | Nodular lymphoma of lymph nodes of inguinal region and leg |
| 95012 | B621800 | Mycosis fungoides of lymph nodes of multiple sites |
| 95016 | B0z1.00 | Malignant neoplasm of Waldeyer's ring |
| 95049 | B616000 | Hodgkin's lymphocytic depletion of unspecified site |
| 95057 | B34y000 | Malignant neoplasm of ectopic site of female breast |
| 95058 | B600700 | Reticulosarcoma of spleen |
| 95182 | B308100 | Malignant neoplasm of talus |
| 95323 | B35z000 | Malignant neoplasm of ectopic site of male breast |
| 95338 | B613600 | Hodgkin's, lymphocytic-histiocytic pred intrapelvic nodes |
| 95378 | B561200 | Secondary and unspec malig neop diaphragmatic lymph nodes |
| 95421 | B45y.00 | Malignant neoplasm of other specified female genital organ |
| 95429 | B071.00 | Malignant neoplasm of posterior wall of nasopharynx |
| 95458 | B300300 | Malignant neoplasm of nasal bone |
| 95480 | B001100 | Malignant neoplasm of lower lip, lipstick area |
| 95505 | B41y000 | Malignant neoplasm of cervical stump |
| 95545 | B627911 | Maltoma |
| 95629 | B325500 | Malignant melanoma of perineum |
| 95630 | B62x600 | True histiocytic lymphoma |
| 95644 | B241.00 | Malignant neoplasm of heart |
| 95671 | Byu5700 | [X]Malignant neoplasm of peritoneum, unspecified |
| 95715 | B627900 | Mucosa-associated lymphoma |
| 95772 | B051000 | Malignant neoplasm of upper buccal sulcus |
| 95783 | B17yz00 | Malignant neoplasm of specified site of pancreas NOS |
| 95792 | B62zz00 | Lymphoid and histiocytic malignancy NOS |
| 95949 | B621000 | Mycosis fungoides of unspecified site |
| 96003 | B055000 | Malignant neoplasm of junction of hard and soft palate |
| 96094 | B119.00 | Siewert type III adenocarcinoma |
| 96226 | ByuC100 | [X]Malignant neoplasm/overlap lesion/other+ill-defined sites |
| 96379 | B621400 | Mycosis fungoides of lymph nodes of axilla and upper limb |
| 96429 | B470z00 | Malignant neoplasm of undescended testis NOS |
| 96445 | B300B00 | Malignant neoplasm of turbinate |
| 96585 | B32y000 | Overlapping malignant melanoma of skin |
| 96635 | B17y000 | Malignant neoplasm of ectopic pancreatic tissue |
| 96782 | B003z00 | Malignant neoplasm of lower lip, inner aspect NOS |
| 96783 | B005.00 | Malignant neoplasm of commissure of lip |
| 96802 | B11y100 | Malignant neoplasm of posterior wall of stomach NEC |
| 96869 | B071z00 | Malignant neoplasm of posterior wall of nasopharynx NOS |
| 96971 | B20y.00 | Malig neop other site nasal cavity, middle ear and sinuses |
| 97091 | ByuC500 | [X]2ndry malignant neoplasm/bladder+oth+unsp urinary organs |
| 97332 | B213z00 | Malignant neoplasm of laryngeal cartilage NOS |
| 97499 | B118.00 | Siewert type II adenocarcinoma |
| 97530 | B051100 | Malignant neoplasm of lower buccal sulcus |
| 97547 | B551200 | Malignant neoplasm of intrathoracic site NOS |
| 97577 | B602300 | Burkitt's lymphoma of intra-abdominal lymph nodes |
| 97672 | B576z00 | Secondary malig neop of retroperitoneum or peritoneum NOS |
| 97746 | B61z800 | Hodgkin's disease NOS of lymph nodes of multiple sites |
| 97832 | B58y211 | Secondary cancer of the cervix |
| 97863 | B615000 | Hodgkin's disease, mixed cellularity of unspecified site |
| 97875 | B175.00 | Malignant neoplasm, overlapping lesion of pancreas |
| 97996 | B44y.00 | Malignant neoplasm of other site of uterine adnexa |
| 98104 | B23y.00 | Malignant neoplasm of other specified pleura |
| 98142 | B107.00 | Siewert type I adenocarcinoma |
| 98361 | Byu5B00 | [X]Kaposi's sarcoma of other sites |
| 98408 | B313z00 | Malig neop of connective and soft tissue of thorax NOS |
| 98500 | B002200 | Malignant neoplasm of upper lip, mucosa |
| 98537 | B201100 | Malignant neoplasm of tympanic cavity |
| 98596 | ByuD200 | [X]Other types of diffuse non-Hodgkin's lymphoma |
| 98626 | B563100 | Secondary and unspec malig neop supratrochlear lymph nodes |
| 98740 | B000z00 | Malignant neoplasm of upper lip, vermilion border NOS |
| 98813 | B500.00 | Malig neop eyeball excl conjunctiva, cornea, retina, choroid |
| 98840 | B610300 | Hodgkin's paragranuloma of intra-abdominal lymph nodes |
| 98909 | B611100 | Hodgkin's granuloma of lymph nodes of head, face and neck |
| 98911 | B200100 | Malignant neoplasm of nasal conchae |
| 99001 | B002100 | Malignant neoplasm of upper lip, frenulum |
| 99012 | B61z500 | Hodgkin's disease NOS of lymph nodes inguinal region and leg |
| 99015 | B66y.00 | Other monocytic leukaemia |
| 99096 | Byu2300 | [X]Malignant neopl/overlapping les/resp+intrathoracic organs |
| 99185 | B062100 | Malignant neoplasm of glossopalatine fold |
| 99240 | B600z00 | Reticulosarcoma NOS |
| 99257 | B324z00 | Malignant melanoma of scalp and neck NOS |
| 99386 | B073200 | Malignant neoplasm posterior margin nasal septum and choanae |
| 99413 | B67yz00 | Other and unspecified leukaemia NOS |
| 99493 | B002.00 | Malignant neoplasm of upper lip, inner aspect |
| 99511 | B574200 | Secondary malignant neoplasm of ileum |
| 99572 | B312500 | Malignant neoplasm of connective and soft tissue of toe |
| 99621 | B520.00 | Malignant neoplasm of cranial nerves |
| 99887 | B60y.00 | Other specified reticulosarcoma or lymphosarcoma |
| 99896 | B12y.00 | Malignant neoplasm of other specified site small intestine |
| 99913 | B510300 | Malignant neoplasm of globus pallidus |
| 99951 | B60z.00 | Reticulosarcoma or lymphosarcoma NOS |
| 100002 | B062z00 | Malignant neoplasm of tonsillar fossa NOS |
| 100006 | B602200 | Burkitt's lymphoma of intrathoracic lymph nodes |
| 100083 | B546.00 | Neuroblastoma |
| 100144 | B004300 | Malignant neoplasm of lip, oral aspect |
| 100232 | B24y.00 | Malig neop of other site of heart, thymus and mediastinum |
| 100296 | B582100 | Secondary malignant neoplasm of skin of face |
| 100352 | B601500 | Lymphosarcoma of lymph nodes of inguinal region and leg |
| 100423 | B610100 | Hodgkin's paragranuloma of lymph nodes of head, face, neck |
| 100532 | B622z00 | Sezary's disease NOS |
| 100584 | B110000 | Malignant neoplasm of cardiac orifice of stomach |
| 100615 | B626500 | Mast cell malignancy of lymph nodes inguinal region and leg |
| 100721 | B002z00 | Malignant neoplasm of upper lip, inner aspect NOS |
| 100733 | B51yz00 | Malignant neoplasm of other part of brain NOS |
| 100786 | B651000 | Chronic eosinophilic leukaemia |
| 100906 | B00z000 | Malignant neoplasm of lip, unspecified, external |
| 100918 | B073z00 | Malignant neoplasm of anterior wall of nasopharynx NOS |
| 101086 | B520z00 | Malignant neoplasm of cranial nerves NOS |
| 101114 | B627A00 | Diffuse non-Hodgkin's large cell lymphoma |
| 101465 | B62z800 | Unspec malig neop lymphoid/histiocytic of multiple sites |
| 101530 | B616z00 | Hodgkin's disease, lymphocytic depletion NOS |
| 101606 | B662.00 | Subacute monocytic leukaemia |
| 101608 | B4A1100 | Malignant neoplasm of ureteropelvic junction |
| 101662 | B565200 | Secondary and unspec malig neop circumflex iliac LN |
| 101668 | Byu5400 | [X]Malignant neoplasm/peripheral nerves of trunk,unspecified |
| 101700 | B139.00 | Hereditary nonpolyposis colon cancer |
| 101707 | B001z00 | Malignant neoplasm of lower lip, vermilion border NOS |
| 101715 | B616700 | Hodgkin's disease, lymphocytic depletion of spleen |
| 101753 | B03y.00 | Malignant neoplasm of other sites of gum |
| 101778 | B442.00 | Malignant neoplasm of broad ligament |
| 101805 | B507000 | Malignant neoplasm of lacrimal sac |
| 101885 | B241400 | Mesothelioma of pericardium |
| 101907 | B182.00 | Overlapping malign lesion of retroperitoneum and peritoneum |
| 101988 | B060100 | Malignant neoplasm of palatine tonsil |
| 102142 | B013000 | Malignant neoplasm of anterior 2/3 of tongue ventral surface |
| 102145 | B322100 | Malignant melanoma of external auditory meatus |
| 102151 | B060200 | Malignant neoplasm of overlapping lesion of tonsil |
| 102158 | B625200 | Letterer-Siwe disease of intrathoracic lymph nodes |
| 102205 | B072z00 | Malignant neoplasm of lateral wall of nasopharynx NOS |
| 102594 | B627E00 | Diffuse large B-cell lymphoma |
| 102688 | ByuD400 | [X]Other malignant immunoproliferative diseases |
| 102715 | B625000 | Letterer-Siwe disease of unspecified sites |
| 102783 | B651200 | Chronic neutrophilic leukaemia |
| 102949 | B312000 | Malignant neoplasm of connective and soft tissue of hip |
| 103245 | B601700 | Lymphosarcoma of spleen |

Table S1. Medcodes for being on the cancer register

| **medcode** | **readcode** | **readterm** |
| --- | --- | --- |
| 398 | G580.00 | Congestive heart failure |
| 884 | G581.00 | Left ventricular failure |
| 2062 | G58..00 | Heart failure |
| 2906 | G580.11 | Congestive cardiac failure |
| 4024 | G58z.00 | Heart failure NOS |
| 1223 | G58..11 | Cardiac failure |
| 5942 | G581.13 | Impaired left ventricular function |
| 13189 | 662g.00 | New York Heart Association classification - class II |
| 18853 | 662f.00 | New York Heart Association classification - class I |
| 19066 | 662h.00 | New York Heart Association classification - class III |
| 5255 | G581000 | Acute left ventricular failure |
| 32671 | G580100 | Chronic congestive heart failure |
| 10079 | G580.12 | Right heart failure |
| 9524 | G580.14 | Biventricular failure |
| 17278 | G58z.12 | Cardiac failure NOS |
| 10154 | G580.13 | Right ventricular failure |
| 23707 | G580000 | Acute congestive heart failure |
| 27964 | G582.00 | Acute heart failure |
| 23481 | G581.11 | Asthma - cardiac |
| 27884 | G580200 | Decompensated cardiac failure |
| 51214 | 662i.00 | New York Heart Association classification - class IV |
| 11424 | G580300 | Compensated cardiac failure |
| 22262 | G1yz100 | Rheumatic left ventricular failure |
| 43618 | G581.12 | Pulmonary oedema - acute |
| 12590 | G58z.11 | Weak heart |
| 94870 | G580400 | Congestive heart failure due to valvular disease |
| 101138 | G583.00 | Heart failure with normal ejection fraction |
| 101137 | G583.11 | HFNEF - heart failure with normal ejection fraction |

Table S2. Medcodes for being on the heart failure register

| **medcode** | **readcode** | **readterm** | **Column1** |
| --- | --- | --- | --- |
| 94383 | C10N000 | Secondary diabetes mellitus without complication | |
| 22487 | C10N.00 | Secondary diabetes mellitus | |
| 43857 | C10M.00 | Lipoatrophic diabetes mellitus | |
| 67212 | C10H000 | DM induced by non-steroid drugs without complication | |
| 61122 | C10H.00 | Diabetes mellitus induced by non-steroid drugs | |
| 96506 | C10G000 | Secondary pancreatic diabetes mellitus without complication | |
| 51697 | C10G.00 | Secondary pancreatic diabetes mellitus | |
| 95539 | C10FS00 | Maternally inherited diabetes mellitus | |
| 63690 | C10FR00 | Type 2 diabetes mellitus with gastroparesis | |
| 25591 | C10FQ00 | Type 2 diabetes mellitus with exudative maculopathy | |
| 51756 | C10FP00 | Type 2 diabetes mellitus with ketoacidotic coma | |
| 32627 | C10FN00 | Type 2 diabetes mellitus with ketoacidosis | |
| 85991 | C10FM11 | Type II diabetes mellitus with persistent microalbuminuria | |
| 18390 | C10FM00 | Type 2 diabetes mellitus with persistent microalbuminuria | |
| 60796 | C10FL11 | Type II diabetes mellitus with persistent proteinuria | |
| 26054 | C10FL00 | Type 2 diabetes mellitus with persistent proteinuria | |
| 34450 | C10FK00 | Hyperosmolar non-ketotic state in type 2 diabetes mellitus | |
| 64668 | C10FJ11 | Insulin treated Type II diabetes mellitus | |
| 1407 | C10FJ00 | Insulin treated Type 2 diabetes mellitus | |
| 35385 | C10FH00 | Type 2 diabetes mellitus with neuropathic arthropathy | |
| 59253 | C10FG00 | Type 2 diabetes mellitus with arthropathy | |
| 37806 | C10FF00 | Type 2 diabetes mellitus with peripheral angiopathy | |
| 93727 | C10FE11 | Type II diabetes mellitus with diabetic cataract | |
| 44982 | C10FE00 | Type 2 diabetes mellitus with diabetic cataract | |
| 98723 | C10FD11 | Type II diabetes mellitus with hypoglycaemic coma | |
| 46917 | C10FD00 | Type 2 diabetes mellitus with hypoglycaemic coma | |
| 102201 | C10FC11 | Type II diabetes mellitus with nephropathy | |
| 12640 | C10FC00 | Type 2 diabetes mellitus with nephropathy | |
| 50527 | C10FB11 | Type II diabetes mellitus with polyneuropathy | |
| 18425 | C10FB00 | Type 2 diabetes mellitus with polyneuropathy | |
| 95351 | C10FA11 | Type II diabetes mellitus with mononeuropathy | |
| 62674 | C10FA00 | Type 2 diabetes mellitus with mononeuropathy | |
| 53392 | C10F911 | Type II diabetes mellitus without complication | |
| 47954 | C10F900 | Type 2 diabetes mellitus without complication | |
| 47315 | C10F711 | Type II diabetes mellitus - poor control | |
| 25627 | C10F700 | Type 2 diabetes mellitus - poor control | |
| 49655 | C10F611 | Type II diabetes mellitus with retinopathy | |
| 18496 | C10F600 | Type 2 diabetes mellitus with retinopathy | |
| 12736 | C10F500 | Type 2 diabetes mellitus with gangrene | |
| 91646 | C10F411 | Type II diabetes mellitus with ulcer | |
| 49074 | C10F400 | Type 2 diabetes mellitus with ulcer | |
| 43227 | C10F311 | Type II diabetes mellitus with multiple complications | |
| 65267 | C10F300 | Type 2 diabetes mellitus with multiple complications | |
| 98616 | C10F211 | Type II diabetes mellitus with neurological complications | |
| 34268 | C10F200 | Type 2 diabetes mellitus with neurological complications | |
| 100964 | C10F111 | Type II diabetes mellitus with ophthalmic complications | |
| 47321 | C10F100 | Type 2 diabetes mellitus with ophthalmic complications | |
| 57278 | C10F011 | Type II diabetes mellitus with renal complications | |
| 18777 | C10F000 | Type 2 diabetes mellitus with renal complications | |
| 22884 | C10F.11 | Type II diabetes mellitus | |
| 758 | C10F.00 | Type 2 diabetes mellitus | |
| 95636 | C10ER00 | Latent autoimmune diabetes mellitus in adult | |
| 55239 | C10EQ00 | Type 1 diabetes mellitus with gastroparesis | |
| 97894 | C10EP11 | Type I diabetes mellitus with exudative maculopathy | |
| 22871 | C10EP00 | Type 1 diabetes mellitus with exudative maculopathy | |
| 66145 | C10EN11 | Type I diabetes mellitus with ketoacidotic coma | |
| 40837 | C10EN00 | Type 1 diabetes mellitus with ketoacidotic coma | |
| 62209 | C10EM11 | Type I diabetes mellitus with ketoacidosis | |
| 10692 | C10EM00 | Type 1 diabetes mellitus with ketoacidosis | |
| 102620 | C10EL11 | Type I diabetes mellitus with persistent microalbuminuria | |
| 30294 | C10EL00 | Type 1 diabetes mellitus with persistent microalbuminuria | |
| 30323 | C10EK00 | Type 1 diabetes mellitus with persistent proteinuria | |
| 54008 | C10EJ00 | Type 1 diabetes mellitus with neuropathic arthropathy | |
| 18642 | C10EH00 | Type 1 diabetes mellitus with arthropathy | |
| 93468 | C10EG00 | Type 1 diabetes mellitus with peripheral angiopathy | |
| 100770 | C10EF12 | Insulin dependent diabetes mellitus with diabetic cataract | |
| 49554 | C10EF00 | Type 1 diabetes mellitus with diabetic cataract | |
| 99716 | C10EE12 | Insulin dependent diabetes mellitus with hypoglycaemic coma | |
| 39070 | C10EE00 | Type 1 diabetes mellitus with hypoglycaemic coma | |
| 102163 | C10ED12 | Insulin dependent diabetes mellitus with nephropathy | |
| 10418 | C10ED00 | Type 1 diabetes mellitus with nephropathy | |
| 101311 | C10EC12 | Insulin dependent diabetes mellitus with polyneuropathy | |
| 91943 | C10EC11 | Type I diabetes mellitus with polyneuropathy | |
| 46301 | C10EC00 | Type 1 diabetes mellitus with polyneuropathy | |
| 68105 | C10EB00 | Type 1 diabetes mellitus with mononeuropathy | |
| 99719 | C10EA12 | Insulin-dependent diabetes without complication | |
| 62613 | C10EA11 | Type I diabetes mellitus without complication | |
| 69676 | C10EA00 | Type 1 diabetes mellitus without complication | |
| 97849 | C10E912 | Insulin dependent diabetes maturity onset | |
| 96235 | C10E911 | Type I diabetes mellitus maturity onset | |
| 40682 | C10E900 | Type 1 diabetes mellitus maturity onset | |
| 72702 | C10E812 | Insulin dependent diabetes mellitus - poor control | |
| 35288 | C10E800 | Type 1 diabetes mellitus - poor control | |
| 93875 | C10E712 | Insulin dependent diabetes mellitus with retinopathy | |
| 95343 | C10E711 | Type I diabetes mellitus with retinopathy | |
| 18387 | C10E700 | Type 1 diabetes mellitus with retinopathy | |
| 102112 | C10E611 | Type I diabetes mellitus with gangrene | |
| 69993 | C10E600 | Type 1 diabetes mellitus with gangrene | |
| 98704 | C10E512 | Insulin dependent diabetes mellitus with ulcer | |
| 93878 | C10E511 | Type I diabetes mellitus with ulcer | |
| 18683 | C10E500 | Type 1 diabetes mellitus with ulcer | |
| 54600 | C10E412 | Unstable insulin dependent diabetes mellitus | |
| 49949 | C10E411 | Unstable type I diabetes mellitus | |
| 43921 | C10E400 | Unstable type 1 diabetes mellitus | |
| 45276 | C10E312 | Insulin dependent diabetes mellitus with multiple complicat | |
| 91942 | C10E311 | Type I diabetes mellitus with multiple complications | |
| 47650 | C10E300 | Type 1 diabetes mellitus with multiple complications | |
| 101735 | C10E212 | Insulin-dependent diabetes mellitus with neurological comps | |
| 42831 | C10E200 | Type 1 diabetes mellitus with neurological complications | |
| 98071 | C10E112 | Insulin-dependent diabetes mellitus with ophthalmic comps | |
| 99311 | C10E111 | Type I diabetes mellitus with ophthalmic complications | |
| 47649 | C10E100 | Type 1 diabetes mellitus with ophthalmic complications | |
| 102946 | C10E012 | Insulin-dependent diabetes mellitus with renal complications | |
| 47582 | C10E000 | Type 1 diabetes mellitus with renal complications | |
| 51261 | C10E.12 | Insulin dependent diabetes mellitus | |
| 12455 | C10E.11 | Type I diabetes mellitus | |
| 1549 | C10E.00 | Type 1 diabetes mellitus | |
| 59991 | C10D.11 | Maturity onset diabetes in youth type 2 | |
| 36695 | C10D.00 | Diabetes mellitus autosomal dominant type 2 | |
| 98392 | C10C.12 | Maturity onset diabetes in youth type 1 | |
| 46624 | C10C.11 | Maturity onset diabetes in youth | |
| 43453 | C10C.00 | Diabetes mellitus autosomal dominant | |
| 36633 | C109K00 | Hyperosmolar non-ketotic state in type 2 diabetes mellitus | |
| 18264 | C109J12 | Insulin treated Type II diabetes mellitus | |
| 37648 | C109J11 | Insulin treated non-insulin dependent diabetes mellitus | |
| 18278 | C109J00 | Insulin treated Type 2 diabetes mellitus | |
| 711 | C10..00 | Diabetes mellitus | |

Table S3. Medcodes for being on the diabetes register

| **medcode** | **readcode** | **readterm** |
| --- | --- | --- |
| 33543 | G6X..00 | Cerebrl infarctn due/unspcf occlusn or sten/cerebrl artrs |
| 40758 | G6W..00 | Cereb infarct due unsp occlus/stenos precerebr arteries |
| 39344 | G676000 | Cereb infarct due cerebral venous thrombosis, nonpyogenic |
| 12833 | G668.00 | Right sided CVA |
| 7780 | G667.00 | Left sided CVA |
| 51767 | G666.00 | Pure sensory lacunar syndrome |
| 33499 | G665.00 | Pure motor lacunar syndrome |
| 17322 | G664.00 | Cerebellar stroke syndrome |
| 8443 | G663.00 | Brain stem stroke syndrome |
| 19260 | G662.00 | Posterior cerebral artery syndrome |
| 19280 | G661.00 | Anterior cerebral artery syndrome |
| 18689 | G660.00 | Middle cerebral artery syndrome |
| 6116 | G66..13 | CVA - Cerebrovascular accident unspecified |
| 6253 | G66..12 | Stroke unspecified |
| 1298 | G66..11 | CVA unspecified |
| 1469 | G66..00 | Stroke and cerebrovascular accident unspecified |
| 15788 | G65zz00 | Transient cerebral ischaemia NOS |
| 16507 | G65z100 | Intermittent cerebral ischaemia |
| 55247 | G65z000 | Impending cerebral ischaemia |
| 1895 | G65z.00 | Transient cerebral ischaemia NOS |
| 19354 | G65y.00 | Other transient cerebral ischaemia |
| 50594 | G654.00 | Multiple and bilateral precerebral artery syndromes |
| 44765 | G653.00 | Carotid artery syndrome hemispheric |
| 23465 | G652.00 | Subclavian steal syndrome |
| 21118 | G651000 | Vertebro-basilar artery syndrome |
| 33377 | G651.00 | Vertebral artery syndrome |
| 5268 | G650.11 | Insufficiency - basilar artery |
| 23942 | G650.00 | Basilar artery syndrome |
| 2417 | G65..13 | Vertebro-basilar insufficiency |
| 1433 | G65..12 | Transient ischaemic attack |
| 3132 | G65..11 | Drop attack |
| 504 | G65..00 | Transient cerebral ischaemia |
| 26424 | G64z400 | Infarction of basal ganglia |
| 10504 | G64z300 | Right sided cerebral infarction |
| 9985 | G64z200 | Left sided cerebral infarction |
| 5185 | G64z111 | Lateral medullary syndrome |
| 47642 | G64z100 | Wallenberg syndrome |
| 25615 | G64z000 | Brainstem infarction |
| 5602 | G64z.12 | Cerebellar infarction |
| 15252 | G64z.11 | Brainstem infarction NOS |
| 3149 | G64z.00 | Cerebral infarction NOS |
| 27975 | G641000 | Cerebral infarction due to embolism of cerebral arteries |
| 34758 | G641.11 | Cerebral embolus |
| 15019 | G641.00 | Cerebral embolism |
| 36717 | G640000 | Cerebral infarction due to thrombosis of cerebral arteries |
| 16517 | G640.00 | Cerebral thrombosis |
| 6155 | G64..13 | Stroke due to cerebral arterial occlusion |
| 569 | G64..12 | Infarction - cerebral |
| 5363 | G64..11 | CVA - cerebral artery occlusion |
| 8837 | G64..00 | Cerebral arterial occlusion |
| 24446 | G63y100 | Cerebral infarction due to embolism of precerebral arteries |
| 23671 | G63y000 | Cerebral infarct due to thrombosis of precerebral arteries |
| 51326 | G63y.00 | Other precerebral artery occlusion |
| 63830 | G63..12 | Stenosis of precerebral arteries |
| 57495 | G63..11 | Infarction - precerebral |
| 45781 | G63..00 | Precerebral arterial occlusion |
| 3535 | G61z.00 | Intracerebral haemorrhage NOS |
| 19201 | G61X100 | Right sided intracerebral haemorrhage, unspecified |
| 28314 | G61X000 | Left sided intracerebral haemorrhage, unspecified |
| 31060 | G61X.00 | Intracerebral haemorrhage in hemisphere, unspecified |
| 57315 | G618.00 | Intracerebral haemorrhage, multiple localized |
| 30045 | G616.00 | External capsule haemorrhage |
| 62342 | G615.00 | Bulbar haemorrhage |
| 7912 | G614.00 | Pontine haemorrhage |
| 13564 | G613.00 | Cerebellar haemorrhage |
| 46316 | G612.00 | Basal nucleus haemorrhage |
| 40338 | G611.00 | Internal capsule haemorrhage |
| 31595 | G610.00 | Cortical haemorrhage |
| 18604 | G61..12 | Stroke due to intracerebral haemorrhage |
| 6960 | G61..11 | CVA - cerebrovascular accid due to intracerebral haemorrhage |
| 5051 | G61..00 | Intracerebral haemorrhage |

Table S4. Medcodes for being on the stroke register

| **medcode** | **readcode** | **readterm** |
| --- | --- | --- |
| 794 | H32..00 | Emphysema |
| 998 | H3...11 | Chronic obstructive airways disease |
| 1001 | H3...00 | Chronic obstructive pulmonary disease |
| 3243 | H31..00 | Chronic bronchitis |
| 5710 | H3z..00 | Chronic obstructive airways disease NOS |
| 5798 | H312000 | Chronic asthmatic bronchitis |
| 5909 | H312011 | Chronic wheezy bronchitis |
| 9876 | H38..00 | Severe chronic obstructive pulmonary disease |
| 10802 | H37..00 | Moderate chronic obstructive pulmonary disease |
| 10863 | H36..00 | Mild chronic obstructive pulmonary disease |
| 10980 | H322.00 | Centrilobular emphysema |
| 11150 | H311.00 | Mucopurulent chronic bronchitis |
| 12166 | H3y..00 | Other specified chronic obstructive airways disease |
| 14798 | H312100 | Emphysematous bronchitis |
| 15157 | H31z.00 | Chronic bronchitis NOS |
| 15626 | H310000 | Chronic catarrhal bronchitis |
| 16410 | H32yz00 | Other emphysema NOS |
| 23492 | H320z00 | Chronic bullous emphysema NOS |
| 24248 | H313.00 | Mixed simple and mucopurulent chronic bronchitis |
| 25603 | H310.00 | Simple chronic bronchitis |
| 26125 | H312300 | Bronchiolitis obliterans |
| 26306 | H320.00 | Chronic bullous emphysema |
| 27819 | H312.00 | Obstructive chronic bronchitis |
| 33450 | H32z.00 | Emphysema NOS |
| 37247 | H3z..11 | Chronic obstructive pulmonary disease NOS |
| 37959 | H311100 | Fetid chronic bronchitis |
| 40159 | H311000 | Purulent chronic bronchitis |
| 40788 | H32y.00 | Other emphysema |
| 44525 | H312z00 | Obstructive chronic bronchitis NOS |
| 45089 | H31y100 | Chronic tracheobronchitis |
| 46578 | H321.00 | Panlobular emphysema |
| 56860 | H320000 | Segmental bullous emphysema |
| 59263 | H32y111 | Acute interstitial emphysema |
| 60188 | H320200 | Giant bullous emphysema |
| 61118 | H310z00 | Simple chronic bronchitis NOS |
| 61513 | H311z00 | Mucopurulent chronic bronchitis NOS |
| 63479 | H32y200 | MacLeod's unilateral emphysema |
| 66043 | H31y.00 | Other chronic bronchitis |
| 67040 | H3y..11 | Other specified chronic obstructive pulmonary disease |
| 68066 | H31yz00 | Other chronic bronchitis NOS |
| 68662 | H320100 | Zonal bullous emphysema |
| 70787 | H32y100 | Atrophic (senile) emphysema |
| 92955 | H32y000 | Acute vesicular emphysema |
| 93568 | H39..00 | Very severe chronic obstructive pulmonary disease |
| 99536 | H320300 | Bullous emphysema with collapse |

Table S5. Medcodes for being on the COPD register
